# Sesquiterpene Synthase‐Catalysed Formation of a New Medium‐Sized Cyclic Terpenoid Ether from Farnesyl Diphosphate Analogues

**DOI:** 10.1002/cbic.201800218

**Published:** 2018-07-16

**Authors:** Florence Huynh, Daniel J. Grundy, Robert L. Jenkins, David J. Miller, Rudolf K. Allemann

**Affiliations:** ^1^ School of Chemistry Cardiff University Main Building Park Place Cardiff CF10 3AT UK

**Keywords:** biocatalysis, enzyme catalysis, ethers, ring systems, synthases, terpenoids

## Abstract

Terpene synthases catalyse the first step in the conversion of prenyl diphosphates to terpenoids. They act as templates for their substrates to generate a reactive conformation, from which a Mg^2+^‐dependent reaction creates a carbocation–PP_i_ ion pair that undergoes a series of rearrangements and (de)protonations to give the final terpene product. This tight conformational control was exploited for the (*R*)‐germacrene A synthase– and germacradien‐4‐ol synthase–catalysed formation of a medium‐sized cyclic terpenoid ether from substrates containing nucleophilic functional groups. Farnesyl diphosphate analogues with a 10,11‐epoxide or an allylic alcohol were efficiently converted to a 11‐membered cyclic terpenoid ether that was characterised by HRMS and NMR spectroscopic analyses. Further experiments showed that other sesquiterpene synthases, including aristolochene synthase, δ‐cadinene synthase and amorphadiene synthase, yielded this novel terpenoid from the same substrate analogues. This work illustrates the potential of terpene synthases for the efficient generation of structurally and functionally novel medium‐sized terpene ethers.

Terpenoids are the most diverse class of natural products and possess astounding complexity in structure and biological activity. They all arise from a small pool of isoprenoid diphosphate precursors. Amongst the tens of thousands of known terpenoids are primary metabolites such as carotenoids and ubiquinones as well as a large array of secondary metabolites that act, for example, as semiochemicals, pheromones, antioxidants, phytoalexins and cytotoxins.^1^ The structural complexity of these compounds presents a formidable challenge for organic chemistry, and the synthetic generation of terpenoid variants is often difficult. For example, most active analogues of the antimalarial and anticancer drugs artemisinin^2^ and paclitaxel^3^ only have modifications at the lactone group or the amino acid side chain, as other changes are synthetically difficult to introduce. Moreover, terpene hydrocarbons are often heat sensitive and unstable under acidic conditions, and their chemical synthesis is cumbersome.^1^ In the past, great efforts have been made to overcome the challenging total syntheses of such complex ring systems, but the synthesis of terpenoids does not generally compete with direct extraction from natural sources.^4^ An alternative approach to the synthesis of terpene‐derived products exploits a detailed understanding of terpene chemistry^5^ and uses biotransformations to convert substrate analogues to functionalised terpenoids.

Terpene synthases are divided into two classes depending on the pathway used to form the initial carbocation. Class I synthases use the Mg^2+^‐binding motif to ionise the substrate and form an allyl cation. In contrast, class II synthases form the initial carbocation by protonation of the distal double bond or an epoxide derivative thereof.5a Class I terpene synthases comprise a mostly hydrophobic active site, surrounded by an α‐helical barrel with two Mg^2+^‐binding motifs at the entrance.5a A large body of work on probing the chemical steps by using substrate analogues,^6^ mutagenesis,^7^ putative reaction intermediates^8^ and X‐ray crystallography5a, 9 combined with computational approaches5c, 10 has provided a detailed picture of the mechanisms, by which these enzymes catalyse their reactions.5a, 5b Co‐crystal structures of aristolochene synthase with various substrate analogues, Mg^2+^ and PP_i_,9c in conjunction with molecular modelling,10a revealed the detailed physical steps that lead to the generation of the reactive Michaelis complex. The synthase first binds one Mg^2+^ ion and then farnesyl diphosphate (FDP, **1**); two more Mg^2+^ ions follow, and this closes the active site, forming the Michaelis complex.10a Coordination of the diphosphate by the Mg^2+^ ions triggers the generation of a farnesyl cation (**2**) which is chaperoned by the active‐site contour through a series of electrophilic ring closures and rearrangements. Quenching of the final carbocation either by proton loss or by nucleophilic capture generates the terpenoid product.5a, 5b The active‐site template steers this reaction cascade, distinguishing it from many potentially competing, similar energy pathways with exquisite precision. After catalysis of the initial C−O bond breakage, the role of the enzyme in these chemical steps appears to be largely to act as a template that steers the substrate through a series of reactive conformations within an optimised electrostatic environment.5b, 5c, 10b The substrate adopts a specific conformation in the active site that directs or inhibits site‐selective nucleophilic attack by solvent water.

Harnessing the remarkable catalytic power of terpene synthases to generate terpenoids and evolving them in a predictable and tunable manner can open the door to an expansion of the terpenome. Exploring how terpene synthases control or inhibit the nucleophilic capture of carbocation intermediates has recently become a focus of our research. We have explored how native germacradien‐4‐ol synthase from *Streptomyces citricolor* (GdolS) mediates the nucleophilic capture of the final carbocation by water and have converted a δ‐cadinene synthase (DCS) from *Gossypium arboreum* into a GdolS by targeted site‐directed mutagenesis; here a series of loop movements at the active site allowed water ingress to capture the carbocation rather than making use of a bound water molecule in the closed form of the active site.9b, 11 Moreover, we have investigated how FDP analogues containing nucleophilic functional groups are converted by some sesquiterpene synthases; hence 12‐OH‐FDP was converted directly to dihydroartemisinic aldehyde by the amorphadiene synthase (ADS) from *Artemisia annua* leading to the most concise artemisinin synthesis known and demonstrating that hydroxylated FDPs can act as efficient substrates for terpene synthases.^12^ In the past, we have used (*S*)‐germacrene D synthase (GDS) and (*R*)‐germacrene A synthases (GAS; both from *Solidago canadensis*) to generate a pool of modified germacrenes^13^ from a range of fluorinated and methylated FDP analogues. One of these products is a potent attractant of grain aphids and a potential crop protection agent.^14^ The efficiency of these transformations could be improved dramatically by using segmented‐flow methods.^13^, ^15^ Using the understanding of terpene synthase chemistry gained from this and others’ previous work, we surmised that this could be done by introducing a nucleophile into a substrate analogue to intercept a known carbocationic configuration generated through the templating effect of the enzyme. A simple example to illustrate the proof of concept would be to intercept the germacryl cation that is formed initially by 1,10‐terpene cyclases (i.e., those that catalyse an initial 1,10 ring closure of the FDP substrate, vide infra).

Herein, we describe the use of two 1,10‐cyclases, namely GdolS and GAS to synthesise an unnatural cyclic ether terpenoid from two synthetic oxygenated FDP analogues. The former was chosen as an example of this chemoenzymatic intramolecular capture as it is known naturally to use nucleophilic quenching to capture the final carbocation during its catalytic cycle. The latter is a hydrocarbon synthase to exemplify that this can also be done by both types of sesquiterpene synthase. Both are mechanistically simple with no subsequent cyclisations of the 1,10‐cyclisation product (Scheme 1) and hence keep other potential active‐site variables to a minimum. We also show that several other sesquiterpene synthases are capable of converting these substrates to the same cyclic ether.

**Scheme 1:**
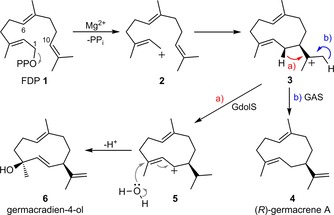
Reactions catalysed by GAS and GdolS with the natural substrate **1**.

Allylic alcohol‐ and epoxide‐containing FDP analogues **7** and **8** were chosen as suitable substrates for this investigation as it was hypothesised that they would efficiently be converted to cyclic ether **10** (Scheme 2). During GAS and GdolS catalysis (Scheme 1), the initially formed farnesyl cation **2** is attacked by the C10=C11 double bond to generate germacryl cation **3**,^16^ so that the alcohol oxygen atom in **7** or the epoxide in **8** (Scheme 2) is ideally placed to intercept the carbocation in intermediate **9** or **11**, respectively. Proton loss from **9** would then furnish cyclic ether **10**. Similarly, cyclisation of epoxy cation **11** should lead to carbocation **12**. Deprotonation from C12 of **12**, as is seen with GAS,^17^ should yield **10**. Although this was considered the most likely outcome, alternative products such as **13**, **14**, or **15** (Scheme 2) had to be ruled out experimentally. Cleavage of the C10−O bond following nucleophilic attack at C1 with final proton loss would give the 12‐membered cyclic ether **15**; alternatively, a [1,2]‐methyl shift from C11 followed by proton loss might result in **13** or **14**.

**Scheme 2:**
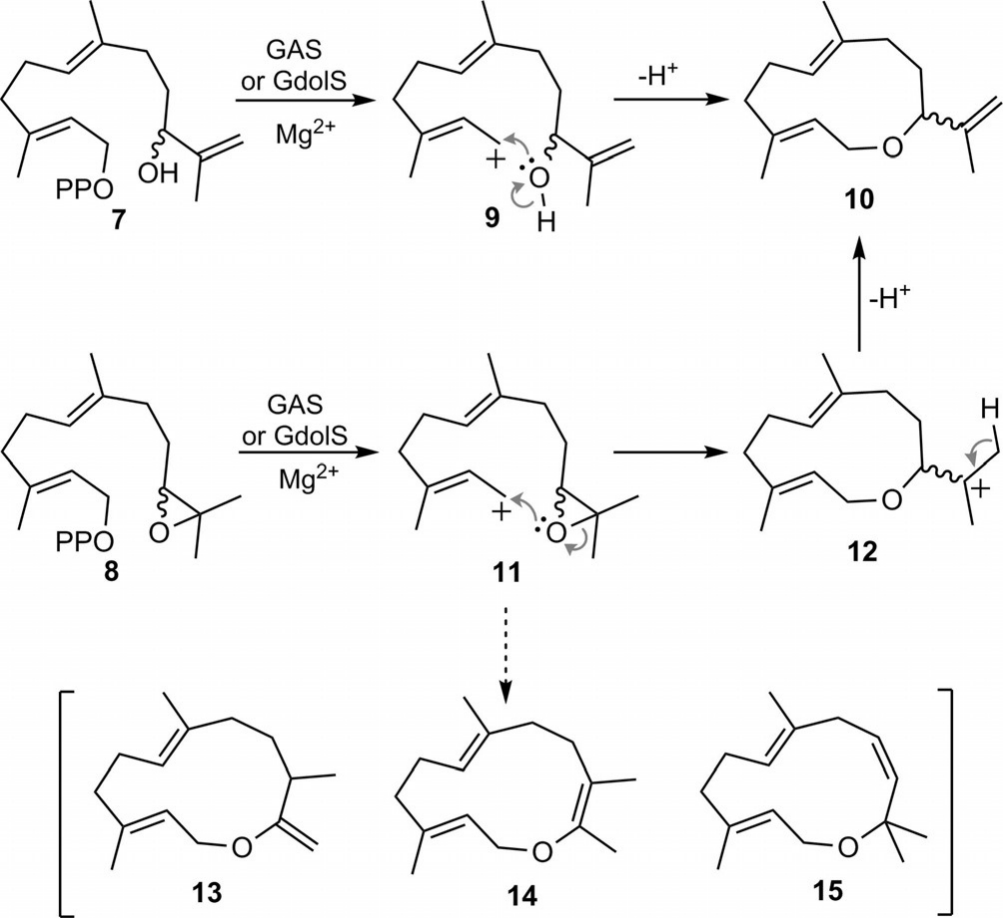
Proposed reaction mechanism for the GAS‐ and GdolS‐catalysed conversions of **7** and **8** to the medium‐sized terpene ether **10**.

Synthetic cDNAs for GAS and GdolS were overexpressed in *Escherichia coli*. The resulting enzymes were purified as previously described9b, 18 and incubated on an analytical scale with synthetically produced **7** and **8** (for synthetic details see Section S2 in the Supporting Information). The pentane‐extractable products were analysed by GC‐MS (Figure 1 and Supporting Information). From the allylic alcohol **7**, GdolS generated a single product with a molecular ion of *m*/*z* 220 (Figures S1 and S2). GAS produced the same product as only 50 % of the pentane‐extractable products with the remaining 50 % comprising a mixture of unidentified products (Figure S3). Incubation of epoxide **8** with GdolS and GAS also gave the same major compound. However, a minor second product was formed by GdolS (Figure 1). The major product for both enzymes showed the same fragmentation pattern (Figures S4 and S5) in the mass spectrum, and co‐elution of all four product mixtures confirmed an identical major product for each reaction (Figure S3). The major product was characterised as compound **10** (vide infra). The minor product (≈10 %) produced by GdolS from **8** was identified by GC‐MS as the epoxide derivative of (*E*)‐β‐farnesene **16** after comparison with a sample generated by conversion of **8** with (*E*)‐β‐farnesene synthase from *Mentha* x *piperita* (EBFS; Figure 1).^19^ As both analogues proved to be substrates, other sesquiterpene synthases were tested. Aristolochene synthase from *Penicillium roqueforti* produced compound **10** as well as an unknown side product from both **7** and **8**. On the other hand, DCS was only able to turn over **8** to **10**, whereas ADS only accepted **7** to produce **10** (Figure S8). To estimate the efficiency with which GdolS turned over **8**, competitive incubations of **8** and **1** with GdolS were performed and analysed by GC‐MS to follow the relative production of **6** and **10**. Incubations of 3 μm enzyme with 0.125 mm **8** and 25 μm FDP led to similar turnovers of the two substrates. This result suggests that turnover for the conversion of **8** by GdolS is about 20 % of that of the natural substrate **1**. No conversion was detected for any substrate in negative controls in which enzyme was absent. Moreover, to demonstrate that this is a specific templating effect of terpene synthases, **8** was incubated with alkaline phosphatase from bovine intestinal mucosa (Sigma–Aldrich) as a positive control experiment. No formation of any cyclised product was observed with only 10,11‐epoxy farnesol, that is, simple hydrolysis product was detected in the pentane‐extractable products only (Figures S9–S10).

**Figure 1:**
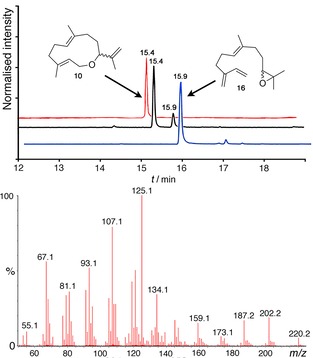
Top: Total ion chromatograms of the pentane‐extractable products from incubations of **8** with GAS (**—**), GdolS (**—**) and EBFS (**—**). Bottom. Mass spectrum of the major product (**10**) eluting at 15.4 min.

To confirm the structure of the major product **10**, a preparative‐scale incubation of **8** with GdolS was performed. A colourless oil was isolated from the incubation of 60 mg of **8** (ESI) in 41 % yield, which is a typical value for natural terpenoids produced from FDP in batch processes.^13^ Unoptimised segmented‐flow procedures, which improve the extraction of the hydrophobic product from the aqueous phase, improved the yield to 75 %.^13^, ^15^ Both substrates (**7** and **8**) were prepared and used in racemic form, and the products were analysed by GC on a chiral stationary‐phase and found to be a mixture of enantiomers with a ratio of 48:52 (Figures S6 and S7). The slight deviation from a racemate in the batch‐generated product arises from different conversion rates of the two enantiomers, as one might expect. This was reinforced when performed in flow (Figure S11). The reaction in flow was performed over 1 h, and a clearly faster turnover of one enantiomer occurred, whereas the batch incubation took several days and led to loss of the initial enantioselectivity seen in flow. Initial ^1^H NMR spectroscopic analysis of the batch product at room temperature in CDCl_3_ showed broad, poorly defined signals in some areas of the spectrum; this hindered a full assignment of the spectrum (Figure 2). Slow exchange on the NMR timescale at room temperature has been observed previously for similar medium‐sized ring systems;^20^ hence variable‐temperature NMR spectra were measured between −50 °C and +50 °C. The structure was successfully elucidated at +50 °C. Full assignments of the ^1^H and ^13^C NMR spectra are given in the Table S1, they confirmed **10** as the major product generated by GdolS from **8**.

**Figure 2:**
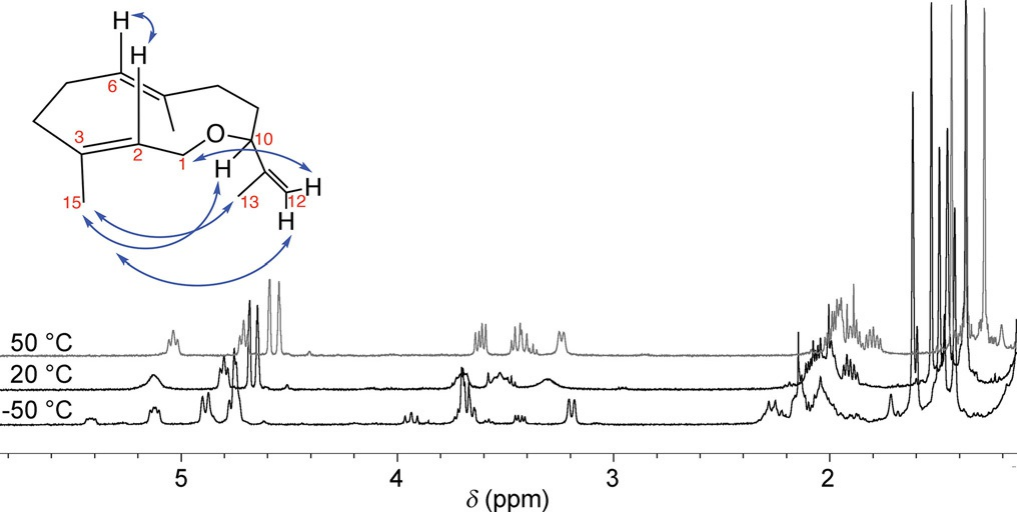
^1^H NMR spectra (400 MHz, CDCl_3_) of **10** at −50, +20 and +50 °C. Inset: observed NOEs for **10**.

At −50 °C, two conformations were apparent: for example, the signal at *δ*_H_=5.32 ppm, corresponding to the proton on C2, split into two resonances at *δ*_H_=5.19 and 5.48 ppm (Figure 2) in an approximately 2:1 ratio. Resonances for the minor conformation of **10** also appeared at −50 °C in all other regions of the spectrum, particularly clearly for the protons on C1 and C10 (*δ*_H_=3.2–4.0 ppm) and in the alkyl region. A NOESY spectrum at −50 °C showed several distinct NOEs (Figure 2 and the Supporting Information), thus allowing some conformational restraints to be applied. Close proximity was apparent between the protons on C2 and C6, C13 and C15, and C10 and C12. Hence, the major conformation of (*R*)‐**10** is the down–down form, in which C14 and C15 are on the same side of the ring system (Figure 3), with (*S*)‐**10** being the mirror image. NOEs were not detectable for the minor isomer; however, the most significant changes in the ^1^H NMR spectrum, as temperature decreased, corresponded to the protons on C1 and C2, thus suggesting that the minor conformer is the up–down conformation (Figure 3), in which the methyl groups are on opposite sides of the ten‐membered ring. These two alkyl groups change position most during the conformational transition.

**Figure 3:**
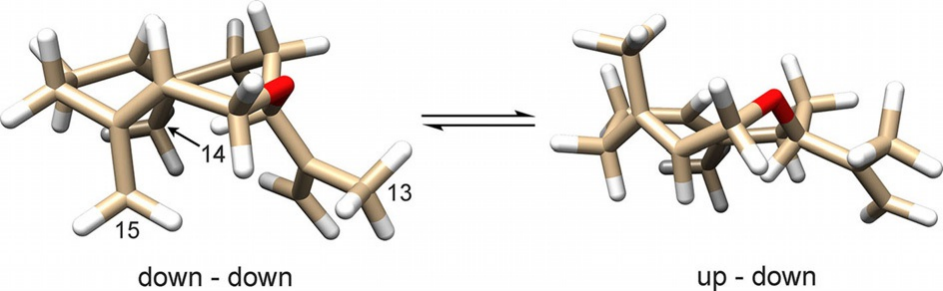
Proposed conformational equilibrium at −50 °C between the major down–down (CH_3_‐14 and 15 compared) conformer of (*R*)‐**10** and the minor up–down form.

The application of biologically active molecules is often dependent on the availability of an efficient and economic synthesis for their production. Terpene synthases can potentially play key roles in the synthesis of blockbusting terpenoids and are already employed in engineered fermentation platforms.^21^ However, such systems are still largely limited to the production of natural terpenoids as they rely on the extended metabolic pathways for FDP synthesis from simple primary metabolites such as acetyl‐CoA.1a The goal to develop biotechnological platforms to generate novel oxygenated terpenoids in a programmable and bespoke manner relies on a demonstration that terpene synthases can generate novel products in vitro. Although there are two examples in the literature in which unnatural heterocycles have been generated by terpene synthases,^22^ and of course many examples of abortive products generated in mechanistic investigations of substrate analogues,^5^, ^6^, ^7^ our work shows for the first time that a novel terpenoid heterocycle can be generated by knowledge‐based design and conversion of a substrate analogue, thereby opening up the use of terpene synthases to the preparation of complex chiral organic compounds with potentially novel activities.

## Conflict of interest

*The authors declare no conflict of interest*.

## Supporting information

As a service to our authors and readers, this journal provides supporting information supplied by the authors. Such materials are peer reviewed and may be re‐organized for online delivery, but are not copy‐edited or typeset. Technical support issues arising from supporting information (other than missing files) should be addressed to the authors.

Supplementary
