# Supplementary material for: Sesquiterpene Synthase‐Catalysed Formation of a New Medium‐Sized Cyclic Terpenoid Ether from Farnesyl Diphosphate Analogues
Source: Chembiochem. 2018 Jul 16;19(17):1834–8. doi: 10.1002/cbic.201800218 (PMC6334173; doi:10.1002/cbic.201800218)
Supplement: Supplementary file 1 — Supplementary [file CBIC-19-1834-s001.pdf]

CHEMBIOCHEM

Supporting Information

Sesquiterpene Synthase-Catalysed Formation of a New Medium-Sized Cyclic Terpenoid Ether from Farnesyl Diphosphate Analogues

Florence Huynh, Daniel J. Grundy, Robert L. Jenkins, David J. Miller, and Rudolf K. Allemann*[a]

cbic_201800218_sm_miscellaneous_information.pdf

Table of Contents

1. General methods and materials. SI1
2. Enzyme preparation and purification. SI1
3. Synthetic Procedures. SI2
4. Analytical Incubation of enzymes with isoprenyl diphosphates. SI5
5. Preparative-scale enzymatic incubation SI5
6. GC-MS data SI6
7. NMR data and spectra SI14
8. References SI23

1. General methods and materials

A prestained protein size marker (14.4-116.0) kDa was used to identify proteins by 12% SDS-gel. The Amicon-YM30 membranes were used for protein concentration. For synthetic procedures, all chemicals and solvents were obtained from Sigma-Aldrich, Acros Chemicals or Alpha Aesar and used without further purification unless otherwise noted. Anhydrous tetrahydrofuran (THF), diethyl ether, toluene and acetonitrile were obtained from a MBraun SPS800 solvent purification system. Dichloromethane, and triethylamine were distilled from calcium hydride and KOH under nitrogen respectively. EtOH was distilled from calcium oxide**.**
^{1}H, ^{31}P and ^{13}C NMR spectra were measured on a Bruker Ultrashield 400 NMR spectrometer or a Bruker Fourier 300 NMR spectrometer and are reported as chemical shifts in parts per million downfield from tetramethylsilane (^{1}H and ^{13}C) or 85% H_3PO_4 (^{31}P), respectively, multiplicity (s = singlet, d = doublet, t = triplet, q = quartet, m = multiplet), coupling (to the nearest 0.5 Hz) and assignment, respectively.

GC-MS analysis of incubation products was performed on a Hewlett Packard 6890 GC instrument fitted with a J&W scientific DB-5MS column (30 m x 0.25 mm internal diameter) and a Micromass GCT Premiere detecting in the range *m/z* 50-800 in the EI^+ mode with scanning once a second with a scan time of 0.9 s. Method 1: The program uses an injection port temperature of 100 °C; split ratio 5:1; initial temperature 50 °C hold 1 min, ramp of 4 °C/min to 150 °C hold 15 min, ramp of 20 °C/min to 250 °C hold 3 min. High-resolution ES^- mass spectra were measured on a Micromass LCT premiere XE spectrometer fitted with a Waters 1525 Micro binary HPLC pump. The purity of purified compounds was judged to be > 95% by TLC and/or GC analyses and NMR spectroscopic analysis. High-resolution ES^- mass spectra were measured on a Micromass LCT premiere XE spectrometer fitted with a Waters 1525 Micro binary HPLC pump.
GC-FID chiral analysis was performed on an Agilent 7890A GC system fitted with a SUPELCO Aztec CHIRALDEX™ B-DM silica capillary column. **GC-FID method I.** A 5 μL sample of compound in dilute pentane solution was injected with a 50:1 split. The oven temperature was held at 70 °C for 3 min and then raised at 5 °C/min to 160 °C and then held for 30 min. **GC-FID method 2.** A 5 μL sample was injected with a 50:1 split. The oven temperature was held at 100 °C for 2 min and then raised at 2 °C/min to 150 °C and then held for 30 min.

Thin layer chromatography was performed on pre-coated aluminium plates of silica G/UV_{254}. TLC visualizations were performed with 4.2% ammonium molybdate and 0.2% ceric sulfate in 5 % H_2SO_4, or 0.1 % berberine hydrochloride in EtOH or UV light at 254 nm. Reverse-phase HPLC was performed on a system comprising of a Dionex P680 pump and a Dionex UVD170U detector unit.

2. Enzyme preparation and purification.

Germacrene A synthase. A codon optimised synthetic gene encoding (*R*)-germacrene A synthase cloned into the pET21d vector was prepared as previously described.[1] *E. coli* BL21(DE3) cells were transformed with 1 μL of plasmid solution. One colony of these cells was added to 100 mL of LB medium containing ampicillin (50 μg/mL) and the culture was allowed to grow at 37 °C with shaking (120 rpm) overnight. A portion (5 mL) of the overnight culture was transferred to each of 6 x 500 mL of LB medium containing the same concentration of ampillicin as before. Cells were incubated at 37 °C with shaking at 120 rpm. When the OD_{600} reached 0.6 - 0.8, isopropyl-β-D-thiogalacto-pyranoside (IPTG) was added (500 μM final concentration) and shaking was continued for 4 hours at 37 °C. Cells were harvested by centrifugation at 5 °C (4200 *g*, 10 min). The supernatant solution was discarded and the pellets were stored at -20 °C.
Pellets were defrosted and resuspended in 50 mL of cell lysis buffer (50 mM Tris-Base, 5 mM EDTA, 5 mM β-mercaptoethanol (βME), pH 8.0). Cells were disrupted by sonication at 5 °C (40 % amplitude for 3 min with 5 s on/10 s off cycles). The resulting suspension was centrifuged at 5 °C (17000 *g* for 30 min) and the supernatant solution was discarded. The protein was recovered from the inclusion bodies by resuspending the pellet in fresh cell lysis buffer (50 mL) and NaOH

(0.1 M) was added to the stirred suspension until the solution became clear (pH ~ 12). This solution was stirred at 4 °C for 30 min and then the pH was lowered to pH 8.0 by dropwise addition of HCl (1.0 M). βME was added (5 mM final concentration) and the solution was stirred for 30 min at 4 °C. This mixture was centrifuged at 5 °C (35000 *g* for 30 min). The protein was found in solution and the pellet was discarded. The supernatant solution was loaded onto a DEAE Fast Flow column (12.5 x 2.5 cm) that had been pre-equilibrated with cell lysis buffer. After loading, the column was washed with 3-5 column volumes (CV) of cell lysis buffer at a flow rate of 10 mL/min. A gradient from 0 to 0.5 M NaCl (4 CV) was followed by a 0.5 to 1 M NaCl gradient (3 CV) and GAS eluted around 0.3 - 0.4 M NaCl. Fractions containing pure protein (SDS-PAGE) were pooled and dialysed overnight against cell lysis buffer (MWCO 12 – 14 kDa). The dialysed fractions were concentrated to a final protein volume of ~ 5mL (AMICON system, YM 30). The concentration of protein was estimated using the method of Bradford.[2]

Germacradien-4-ol synthase. The expression vector containing the gene for GdolS (pET16b-SC1), a generous gift from Professor Yasuo Ohnishi, University of Tokyo[3] and was used to express GdolS in *E. coli* exactly as previously described.[4] Briefly, *E. coli* BL21 (DE3) cells were transformed with pET16b-SC1 and a single transformed colony was used to inoculate 100 mL of LB-medium containing ampicillin (100 mg L^{-1}); this was grown overnight at 37 °C with shaking. This starter culture was used to inoculate LB-medium containing ampicillin (5 mL of culture per 500 mL of LB medium). The cultures were incubated at 37 °C until an OD_{600nm} of 0.6 was reached, at which time gene expression was induced with isopropyl-β-D-1-thiogalactopyranoside (IPTG, 0.2 mM) and the culture allowed to grow for a further 3 h. Cells were harvested by centrifugation (3400 *g*, 10 min), the supernatant was decanted and discarded. Pellets were thawed, resuspended in 25 mL of cell lysis buffer (50 mM Tris, 100 mM NaCl and 10 mM imidazole, pH 8.0) and cells lysed by sonication (5 min, amplitude 40%, pulse 5 s on and 10 s off). The cell debris was discarded after centrifugation (38000 *g*, 30 min) and the supernatant applied to a Ni-NTA affinity column (QIAGEN, 5 mL). Proteins were eluted with a 10 - 500 mM imidazole gradient in lysis buffer. Fractions containing GdolS (purity >95% as judged by SDS-PAGE) were combined, dialyzed against 10 mM Tris, pH 8.0, and concentrated to 10 mL. Protein concentrations were estimated using the method of Bradford[2] and aliquots of GdolS were stored at -20 °C.

Recombinant (*E*)-β-farnesene synthase from *Mentha x piperita*, aristolochene synthase from *Penicillium roqueforti,* amorphadiene synthase from *Artemisia annua* and (+)-δ-cadinene synthase from *Gossypium arboreum* were prepared as previously described.[5]

3. Synthetic Procedures.

(*2E, 6E*)-Farnesyl diphosphate (**1**), used to check protein activity was synthesised from commercial (*2E, 6E*)-farnesol using the method described by Davisson *et al*.[6]

The oxygenated farnesyl diphosphate analogues **7** and **8** were prepared as shown in Scheme S1. Epoxide **19** was generated through a synthetic sequence involving treatment of farnesyl acetate with NBS in wet THF followed by a base catalysed ring closure to form the epoxide.[7] The C10,C11 epoxy-farnesyl diphosphate **8** was then generated from **19** in 66% yield by the method of Davisson *et al.*[6] The allylic alcohol functionality was created by use of an aluminium isopropoxide catalysed epoxide ring opening (65%)[8] and the primary alcohol was selectively converted to a *p*-tosyl leaving group by treatment with *p*-toluenesulfonyl chloride and triethylamine in THF to generate **20**. This was then diphosphorylated by Davisson's methodology to give **7** in 54% yield.[6]

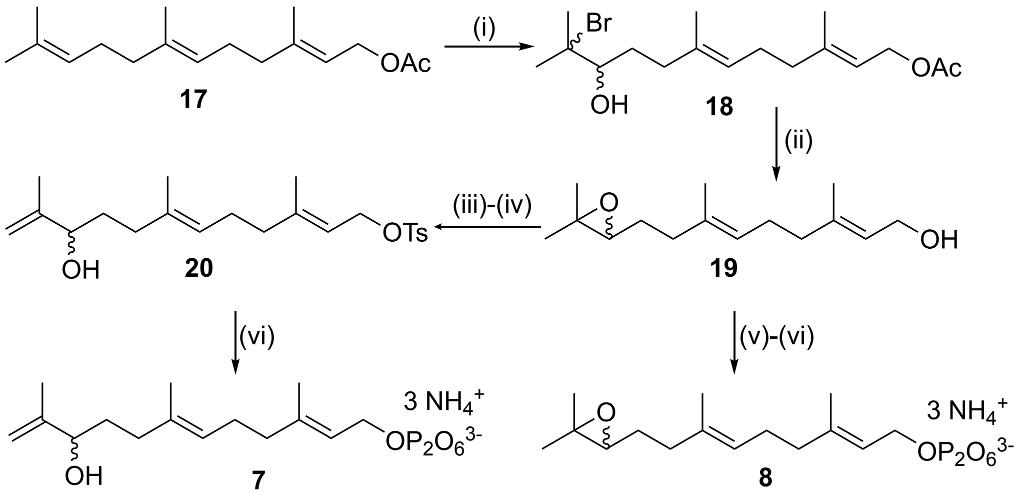

Scheme S1. Reagents and conditions: *(i)* N*-bromosuccinimide, THF, H_2O, 65% (ii) K_2CO_3, MeOH, 92%; (iii) Al(OPr^i)$_3$, toluene, Δ, 65%; (iv) NEt_3,* p*-TsCl, THF; (v) 2,4,6-collidine, MsCl, LiCl, DMF, (vi) $(Bu^n{}_4N)_3HP_2O_7$, CH_3CN then NH_4^+ cation exchange. 54% (2 steps, for* **7***), 66% (2 steps, for* **8***).*

(2*E*,6*E*)-11-Bromo-10-hydroxy-3,7,11-trimethyldodeca-2,6-dien-1-yl acetate (18)

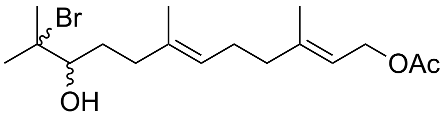

A stirred solution of farnesyl acetate (1.5 g, 5.7 mmol) in THF:H_2O (50 mL, 2:1) was cooled to 0 °C and *N*-bromosuccinimide (1.15 g, 6.45 mmol) was added in portions over 30 min. The reaction was stirred at 0 °C for 2 h before it was allowed to warm to room temperature for a further 2 h. The reaction was diluted with hexane (30 mL) and washed with brine (50 mL), the aqueous layer was further washed with hexane (3 x 30 mL), dried (Na_2SO_4) and solvent removed under reduced pressure. Purification by flash column chromatography on silica (15% EtOAc in hexane, R_f 0.3 in 20%) yielded the title compound as a pale yellow oil (1.15 g, 56%). δ_H (300 MHz, $CDCl_3$) 5.40 – 5.29 (1 H, m, CC*H*CH_2O), 5.19 (1 H, t, *J* 6.0, CC*H*(CH_2)$_2$), 4.59 (2 H, d, *J* 7.0, CCHC*H*$_2$O), 3.97 (1 H, dd, *J* 11.0, 2.0, CBrC*H*OH), 2.41 – 1.90 (8 H, m, 4 x CH_2), 2.06 (3 H, s, C*H*$_3$C(O)), 1.71 (3 H, s, C*H*$_3$), 1.59 (3 H, s, C*H*$_3$), 1.35 (3 H, s, CBrC*H*$_3$), 1.33 (3 H, s, CBrC*H*$_3$); δ_C (151 MHz, $CDCl_3$) 171.3 (*C*(O)CH_3), 142.1 (*C*=CH), 133.7 (*C*=CH), 125.5 (C=*C*H), 118.6 (C=*C*HCH_2O), 70.9 (*C*HOH), 61.5 (C=CH*C*H$_2$O), 39.5, 38.3, 32.2 and 26.7 (CH_2), 26.3 and 26.0 (CBr(*C*H$_3$)$_2$), 21.2 (C(O)*C*H$_3$), 16.6 & 16.0 (C=C*C*H$_3$); HRMS (ES^+, $[M + Na]^+$) found 383.1187, $C_{17}H_{29}O_3BrNa$ requires 383.1198.

(2*E*,6*E*)-9-(3,3-Dimethyloxiran-2-yl)-3,7-dimethylnona-2,6-dien-1-ol (19)

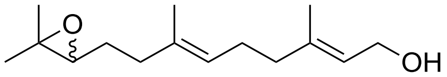

To a stirred solution of **18** (327 mg, 0.91 mmol) in MeOH (20 mL) at room temperature was added K_2CO_3 (248 mg, 1.8 mmol) and the resulting slurry was stirred for 1 h. The mixture was concentrated under reduced pressure and then diluted with hexane (20 mL) and H_2O (20 mL). The aqueous layer separated and washed with hexane (3 x 15 mL). The combined organic fractions were washed with HCl (1 M, 15 mL) and brine, dried (Na_2SO_4) and solvent removed under reduced pressure. Purification by flash column chromatography on a silica plug (30% EtOAc in hexane) yielded the pure product (198 mg, 91%). δ_H (300 MHz, $CDCl_3$) 5.45 – 5.35 (1 H, m, CC*H*CH_2OH), 5.23 – 5.09 (1 H, m, CC*H*CH_2CH_2), 4.14 (2 H, d, *J* 7.0, CCHC*H*2OH), 2.70 (1 H, t, *J* 6.0, C(O)C*H*), 2.20 – 1.98 (6 H, m, 3 x CH_2), 1.67 (3 H, s, CH_3), 1.72 – 1.57 (2 H, m, CH_2), 1.61 (3 H, s, CH_3), 1.30 (3 H, s, CH_3), 1.25 (3 H, s, CH_3); δ_C (75 MHz, $CDCl_3$) 139.5(CH_3*C*=CH), 134.5 (CH_3*C*=CH), 124.6 (C=*C*HCH_2CH_2), 123.7 (C=*C*HCH_2OH), 64.3 (C(O)*C*H), 59.5 (CCH*C*H$_2$OH), 58.6 (*C*(O)CH), 39.5 (CH_2), 36.6 (CH_2), 27.4 (CH_2), 26.2 (CH_2), 25.0 (CH_3), 18.9 (CH_3), 16.4 (CH_3), 16.1 (CH_3); HRMS ($APCI^+$, $[M + Na]^+$) found 261.1828, $C_{15}H_{26}O_2Na$ requires 261.1831.

(2*E*,6*E*)-9-(3,3-Dimethyloxiran-2-yl)-3,7-dimethylnona-2,6-dien-1-yl trisammonium diphosphate (8)

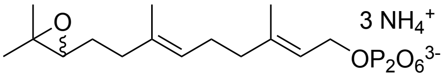

A stirred solution of **19** (65 mg, 0.28 mmol) and 2,4,6-collidine (223 μL, 1.68 mmol) in anhydrous DMF (10 mL) was cooled to 0 °C was added methanesulfonyl chloride (43 μL, 0.56 mmol). The resulting milky solution was stirred for 15 min before the addition of LiCl (47 mg, 1.12 mmol) and then left to stir at 0 °C for a further 2 h. The reaction was quenched with H_2O (5 mL) and washed with hexane (5 x 5 mL). The combined organic extracts were washed with saturated solutions of $CuSO_4$ (25 mL), $NaHCO_3$ (25 mL) and brine (25 mL), then dried over anhydrous $MgSO_4$. After filtration under gravity, the solvent removed under reduced pressure, and dried *in vacuo* for 1 h to yield the intermediate allylic chloride, which was used without further purification.

To a stirred solution of the allylic chloride intermediate in anhydrous acetonitrile was added tris(tetrabutylammonium) hydrogendiphosphate (758 mg, 0.84 mmol) and the resulting mixture was stirred under N_2 for 16 h. The acetonitrile was removed under reduced pressure and the sticky brown residue was dissolved in 2 mL of ion-exchange buffer (25 mM NH_4HCO_3, 2% Pr^iOH), and the resulting cloudy solution was passed slowly through a column of Dowex 50W-X8 cation-exchange resin pre-equilibrated in ion-exchange buffer. Fractions were eluted with the same buffer and analysed by TLC (3:1:1, Pr^iOH:NH_4OH:buffer) and the fractions found to contain product were combined and lyophilised. The white powder was dissolved in 2 mL ion-exchange buffer and purified by reverse-phase HPLC (C18 column, eluting with 10% A for 20 min, then a linear gradient to 60% A over 25 min and finally a linear gradient to 100% A over 5 min.; solvent A: CH_3CN, solvent B: 25 mM NH_4HCO_3, flow rate 5.0 cm^3 min^{-1}, detecting at 220 nm) to give the title compound as a white solid (81 mg, 66%). δ_H (300 MHz, D_2O) 5.29 (1 H, t, $^3J_{H,H}$ = 7.0, C=C*H*CH_2OH), 5.10 (1 H, t, $^3J_{H,H}$ = 6.0, C=C*H*CH_2CH_2), 4.29 (2 H, t, $^3J_{H,H}$ = 6.5, C=CHC*H*$_2$OH), 2.84 (1 H, t, $^3J_{H,H}$ = 6.5, C(O)C*H*), 2.06 – 1.88 (6 H, m, 3 x CH_2), 1.60 – 1.49 (2 H, m, CH_2), 1.54 (3 H, s, CH_3), 1.48 (3 H, s, CH_3), 1.14 (3 H, s, CH_3), 1.12 (3 H, s, CH_3). δ_P (122 MHz, D_2O) - 6.14, -9.83. HRMS (ES^-, $[M - H]^-$) found 397.1174, $C_{15}H_{27}O_8P_2$ requires 397.1181.

(2*E*,6*E*)-3,7,11-Trimethyldodeca-2,6,11-triene-1,10-diol

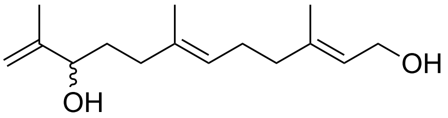

A stirred solution of **19** (113 mg, 0.48 mmol 285 mg, 1.2 mmol) and aluminium isopropoxide (196 mg, 0.96 mmol) in anhydrous toluene (10 mL) was heated to 140 °C in a sealed tube for 16 h. After cooling HCl:EtOAc (5 mL, 1:1, 1 M HCl) was added and stirred for 1 h. The mixture was separated and the aqueous layer extracted with EtOAc (3 x 5 mL) and the combined organic extracts washed with brine, dried ($MgSO_4$), filtered and the solvent was removed under reduced pressure. Purification by column chromatography on silica (40% EtOAc in hexane) yielded the title compound in 65% yield (74 mg, 0.31 mmol). δ_H (300 MHz, $CDCl_3$) 5.49 – 5.33 (1 H, m, C=C*H*CH_2O), 5.24 – 5.10 (1 H, m, C=C*H*$(CH_2)_2$), 4.97 – 4.88 (1 H, m, C*H*H=C(CH_3)CHOH), 4.89 – 4.79 (1 H, m, C*H*H=C(CH_3)CHOH), 4.13 (2 H, d, $^3J_{H,H}$ = 7.0, CCHC*H_2*O), 4.04 (1 H, dd, $^3J_{H,H}$ = 7.5, 5.0, C=CC*H*OH), 2.24 – 2.00 (6 H, m, 3 x CH_2), 1.72 (3 H, s, CH_3), 1.66 (3 H, s, CH_3), 1.69 – 1.60 (2 H, m, CH_2), 1.63 (3 H, s, CH_3); δ_C (151 MHz, $CDCl_3$) 147.6 (C=C), 139.2 (C=C), 135.5 (C=C), 124.9 (C=*C*HCH_2), 124.2 (C=*C*HCH_2OH), 111.0 (*C*H_2=C(CH_3)CHOH), 75.7(C=C*C*HOH), 59.5 (C=CH*C*H_2OH), 39.5, 36.08, 32.9 and 26.0 (*C*H_2), 18.0, 16.1 and 16.0 (*C*H_3); HRMS (ES^-, [M - H_2O - H]$^-$) found 219.1747, $C_{15}H_{23}O$ requires 219.1749.

(2*E*,6*E*)-10-Hydroxy-3,7,11-trimethyldodeca-2,6,11-trien-1-yl trisammonium diphosphate (7)

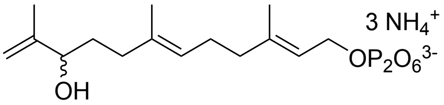

To a stirred solution of (2*E*,6*E*)-3,7,11-trimethyldodeca-2,6,11-triene-1,10-diol (108 mg, 0.45 mmol) and triethylamine (500 μL, 0.9 mmol) in THF (10 mL) was added *para*-toluenesulfonyl chloride (86 mg, 0.45 mmol) and the reaction was left to stir in darkness for 16 h before it was quenched with H_2O (10 mL) and extracted with Et_2O (20 mL). The aqueous layer was further washed with Et_2O (2 x 20 mL) and the combined ethereal extracts washed with saturated solutions of $CuSO_4$ (50 mL), $NaHCO_3$ (50 mL), and brine (50 mL). The organic solution was then dried ($MgSO_4$), filtered under gravity and the solvent was removed under reduced pressure. The tosylate was dried *in vacuo* for 2 h before being used without further purification.
The tosylate intermediate was treated with tris(tetrabutylammonium) hydrogendiphosphate (1.53 mmol, 1.38 g) in anhydrous acetonitrile, and further treated and purified in the same manner as diphosphate **8** to yield the title compound as a white powder in 54% yield (95 mg, 0.24 mmol). δ_H (300 MHz, D_2O) 5.32 (1 H, t, $^3J_{H,H}$ = 6.5, CC*H*CH_2O), 5.09 (1 H, t, $^3J_{H,H}$ = 6.0, CC*H*$(CH_2)_2$), 4.79 (1 H, s, C*H*H=C(CH_3)CHOH), 4.76 (1 H, s, C*H*H=C(CH_3)CHOH), 4.33 (2 H, t, $^3J_{H,H}$ = 6.5, CCHC*H_2*O), 3.93 (1 H, t, $^3J_{H,H}$ = 7.0, C=CC*H*OH), 2.09 – 1.80 (6 H, m, 3 x CH_2), 1.60 – 1.48 (2 H, m, CH_2), 1.58 (3 H, s, CH_3), 1.55 (3 H, s, CH_3), 1.49 (3 H, s, CH_3); δ_P (202 MHz, D_2O) -8.93, -10.35. HRMS (ES^-, [M - H]$^-$) found 397.1193, $C_{15}H_{27}O_8P_2$ requires 397.1181.

(5*E*,9*E*)-5,9-dimethyl-2-(prop-1-en-2-yl)oxacycloundeca-5,9-diene (10)

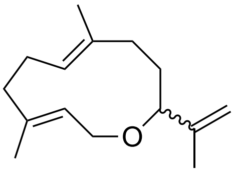

To a gently stirred solution of **8** (30 mg, 67 μmol) in incubation buffer (50 mM Tris, 5 mM β-mercaptoethanol, 5 mM $MgCl_2$, pH 8.0, 450 mL) was added GAS to a final concentration of 3 μM and the mixture was overlaid with pentane (50 mL). The flask was stoppered and stirred gently for 24 h at room temperature then a further portion of **8** (30 mg, 67 μmol) was added into the aqueous layer. After a further 24 h the solution was cooled to 4 °C then stirred for a further 64 h. The pentane layer was separated and the aqueous layer was extracted with pentane (5 x 5 ml), taking care to extract with gentle swirling as shaking generated a thick, intractable emulsion. The pooled organic extracts were then washed with brine (20 ml) before being dried over anhydrous Na_2SO_4, filtered and evaporated carefully under reduced pressure (500 mbar pressure, 25 °C water bath temperature). Purification by preparative thin layer chromatography on silica (5% ether in pentane) gave the title compound as a colourless oil (12.2 mg, 41%). δ_H (400 MHz, $CDCl_3$, 323 K) ^{1}H NMR (400 MHz, $CDCl_3$, 323 K) δ_H 5.32 (1 H, dd, $^3J_{H,H}$ = 9.0 and $^3J_{H,H}$ = 7.0 , C=C*H*CH_2O), 4.98 (1 H, t, $^3J_{H,H}$ = 7.0, C=C*H*CH_2CH_2), 4.88 – 4.84 (1 H, s, C=C*H*H), 4.84 – 4.79 (1H, s, C=CH*H*), 3.89 (1 H, dd, $^2J_{H,H}$ = 12.0, $^3J_{H,H}$ = 7.0 , CHC*H_2*O), 3.70 (1 H, dd, $^2J_{H,H}$ = 9.0, $^3J_{H,H}$ = 7.0, CHC*H_2*O), 3.51 (1 H, m, CH_2OC*H*C=C), 2.32 – 2.02 (6 H, m, 3 x C*H_2*), 1.70 (3 H, s, C*H_3*C=CH_2), 1.68 – 1.52 (2H, m, C*H_2*CHO), 1.64 (3H, s, C*H_3*CC=CHCH_2O), 1.54 (3H, s, C*H_3*C=CHCH_2CH_2). ^{13}C NMR (151 MHz, $CDCl_3$) δ_C (100 MHz, $CDCl_3$, 323 K) 147.0 (*C*=CHH), 139.3 (CH_3*C*=CHCH_2O), 134.6 (CH_3*C*CH_2CH_2CHO), 127.8 (*C*H=CCH_2CH_2), 124.7 (C=*C*HCH_2O), 110.7 (C=*C*H_2), 78.5 (CH_2O*C*HC=CH_2), 62.7 (*C*H_2OCH), 39.7 (*C*H_2CH_2), 39.3 (*C*H_2CH_2), 39.7 (*C*H_2CH_2), 25.1 (*C*H_2CH_2), 17.8 (*C*H_3C=CH_2), 16.1

(CH_3C=$CHCH_2O$), 15.1 ($CH_3CCH_2CH_2$). *m/z* (EI^+) 220.2 (6%, M^+), 202.2 (22), 187.2 (20), 159.1 (15), 147.1 (12), 145.1 (17), 134.1 (32), 125.1 (100), 121.1 (50), 119.1 (40), 93.1 (51), 91.1 (38), 81.1 (33), 79.1 (32), 68.1 (31), 67.1 (57), 55.1 (10). HRMS (EI^+, M^+) found 220.1828, $C_{15}H_{24}O$ requires 220.1827.

4. Analytical Incubation of enzymes with isoprenyl diphosphates

A solution of 1 μM GAS or GdolS and 200 μM isoprenyl diphosphate in incubation buffer (250 μL 50 mM Tris, 5 mM βME, 5 mM $MgCl_2$, pH 8.0) was prepared. The aqueous layer was overlaid with HPLC grade pentane (0.5 mL) and the resulting mixture was gently agitated (6 - 18 h) at 25 °C. The incubations were repeated without enzyme as negative controls. The pentane extracts were then analysed by gas chromatography-mass spectrometry (GC-MS) as described in General Methods (section 2), flow yields were estimated using GC-FID.[9]

5. Preparative-scale enzymatic incubation

Preparative incubations of the farnesyl diphosphate analogues were used for production of milligram quantities of products. Incubations were scaled to a suitable size dependent on the quantity of substrate being used, to a final substrate concentration of 0.3 mM in buffer (50 mM Tris, 5 mM $MgCl_2$, 5 mM βME, pH 8.0) with an enzyme concentration of 3 μM.
Incubation. Buffer was prepared and the enzyme added at room temperature with gentle stirring, 50% of the total isoprenyl diphosphate was added and the incubation was overlaid with pentane (~10% of total incubation volume) and stirred at room temperature for 24 h. The remaining isoprenyl diphosphate substrate was then added and the completed incubation was stirred for a further 12-24 h before it was cooled to 4 °C and stirred for a further 64 h.
Extraction. The pentane overlay was removed and the aqueous layer was further extracted with pentane (5 x 5% total incubation volume) by gentle swirling and slow separation. The combined pentane extracts were washed with brine and solvent was then removed carefully under reduced pressure (500 mbar minimum pressure at 25 °C water bath temperature to avoid loss of volatile product) to yield the terpenoid product as an oil. (experimental details also in section 3)

6. GC-MS data

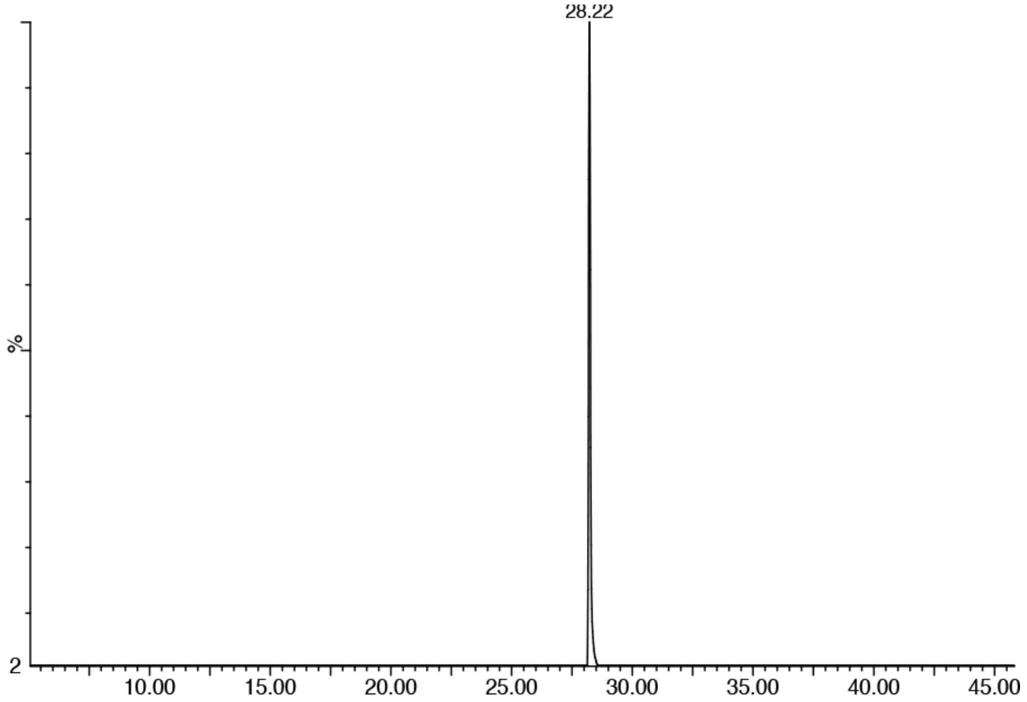

Figure S1. Total ion chromatogram of the pentane extractable products arising from from the incubation of **7** with GdolS.

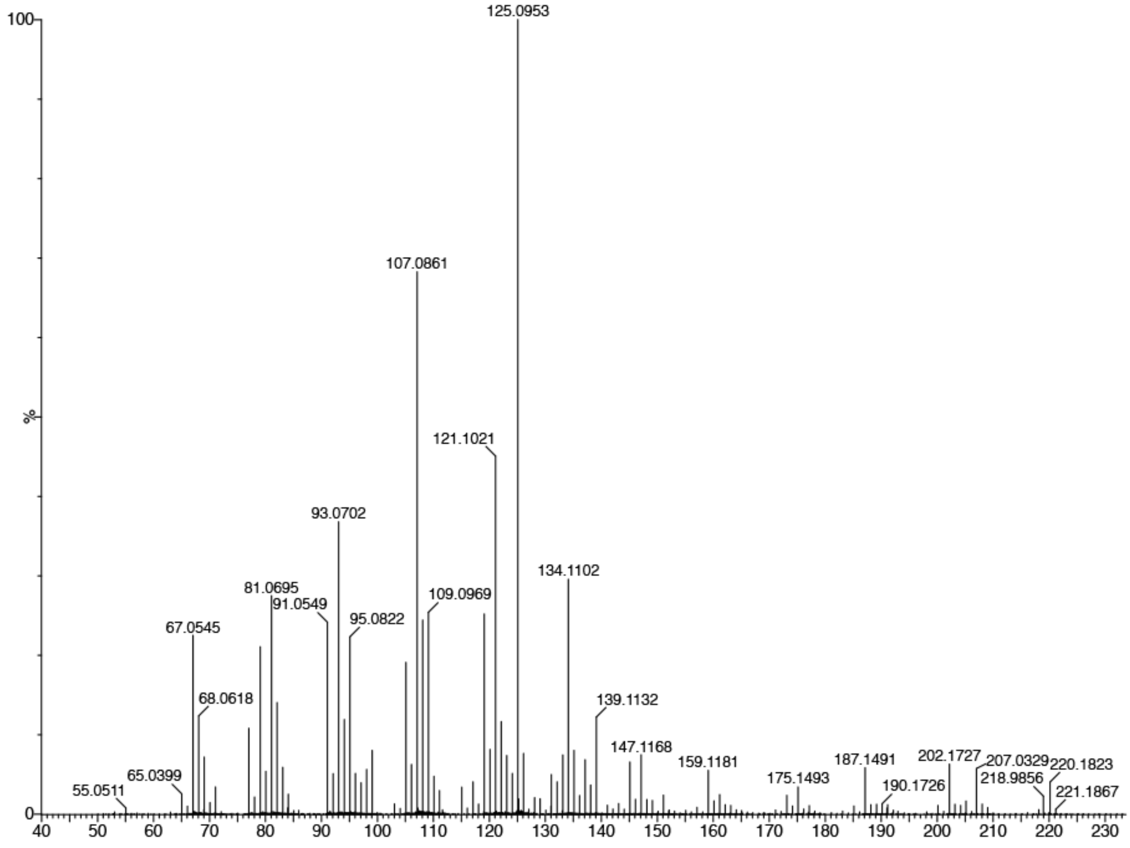

Figure S2. Mass spectrum of the compound eluting at 28.22 min in the gas-chromatogram from the incubation of **7** with GdolS.

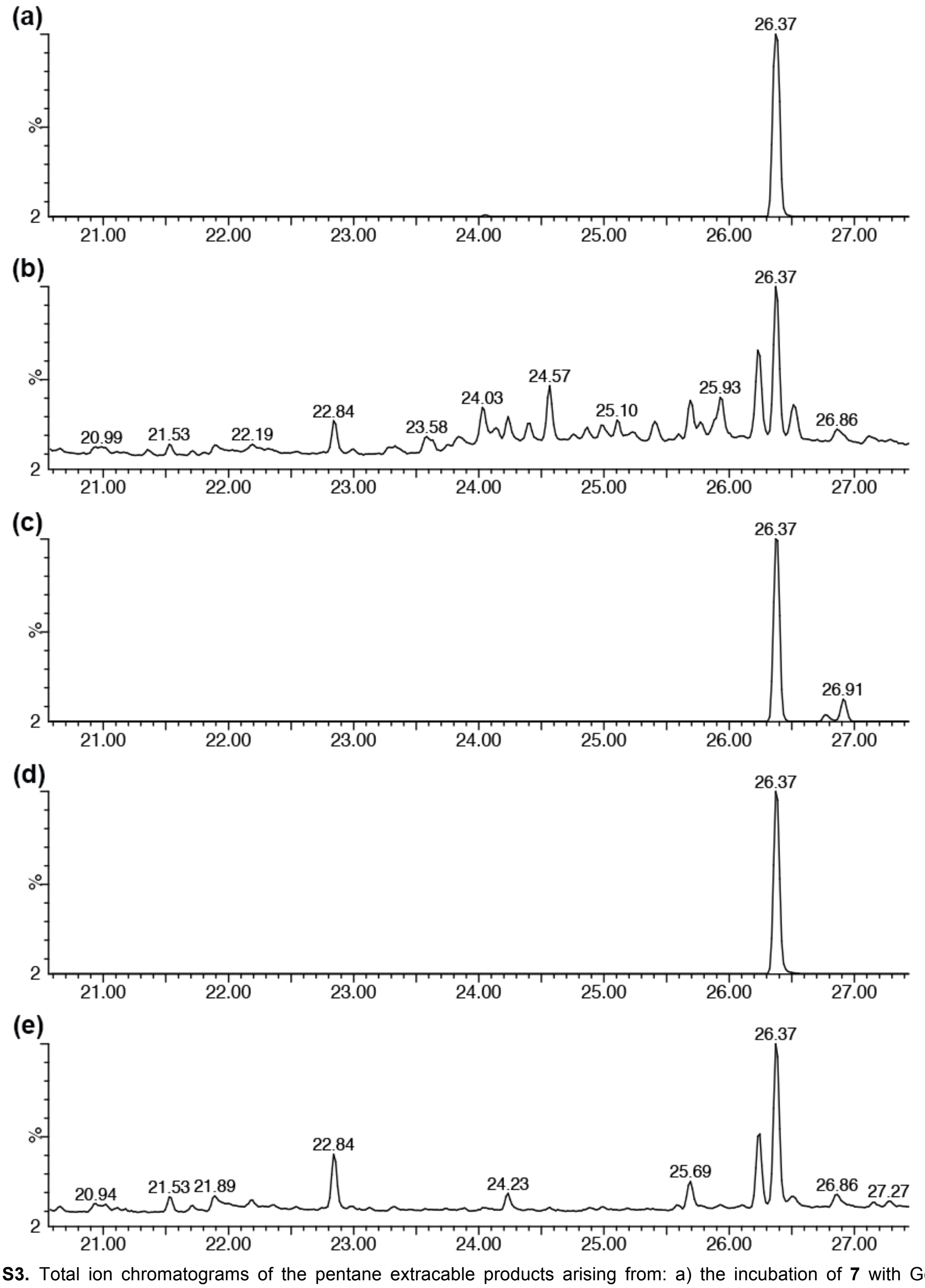

Figure S3. Total ion chromatograms of the pentane extracable products arising from: a) the incubation of **7** with GdolS, b) the incubation of **8** with GdolS, c) the incubation of **7** with GAS and d) from the incubation of **8** with GAS, e) from the mixture of four incubations – GAS with **7**, GAS with **8**, GdolS with **7** and GdolS with **8**. The same product **10** coelutes for all four incubations. Unidentified products at 24.23, 25.69, 26.24, 27.27, 28.16 and 28.84 min arise from incubation of **7** with GAS.

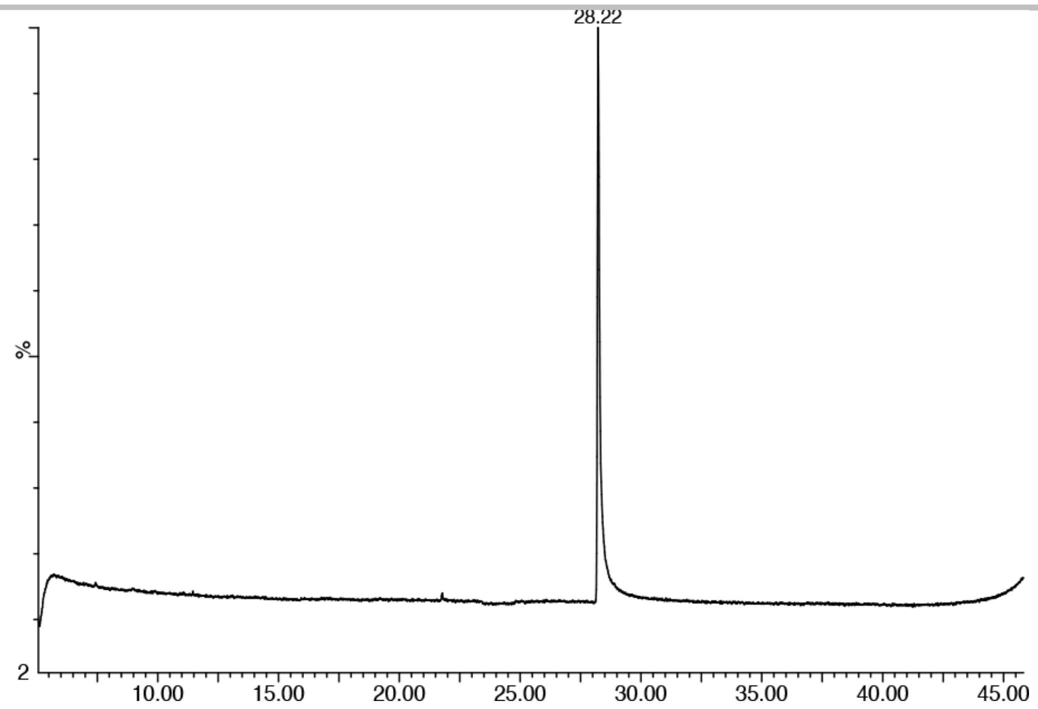

Figure S4. Total ion chromatogram of the pentane extractable products from the incubation of **8** with GAS.

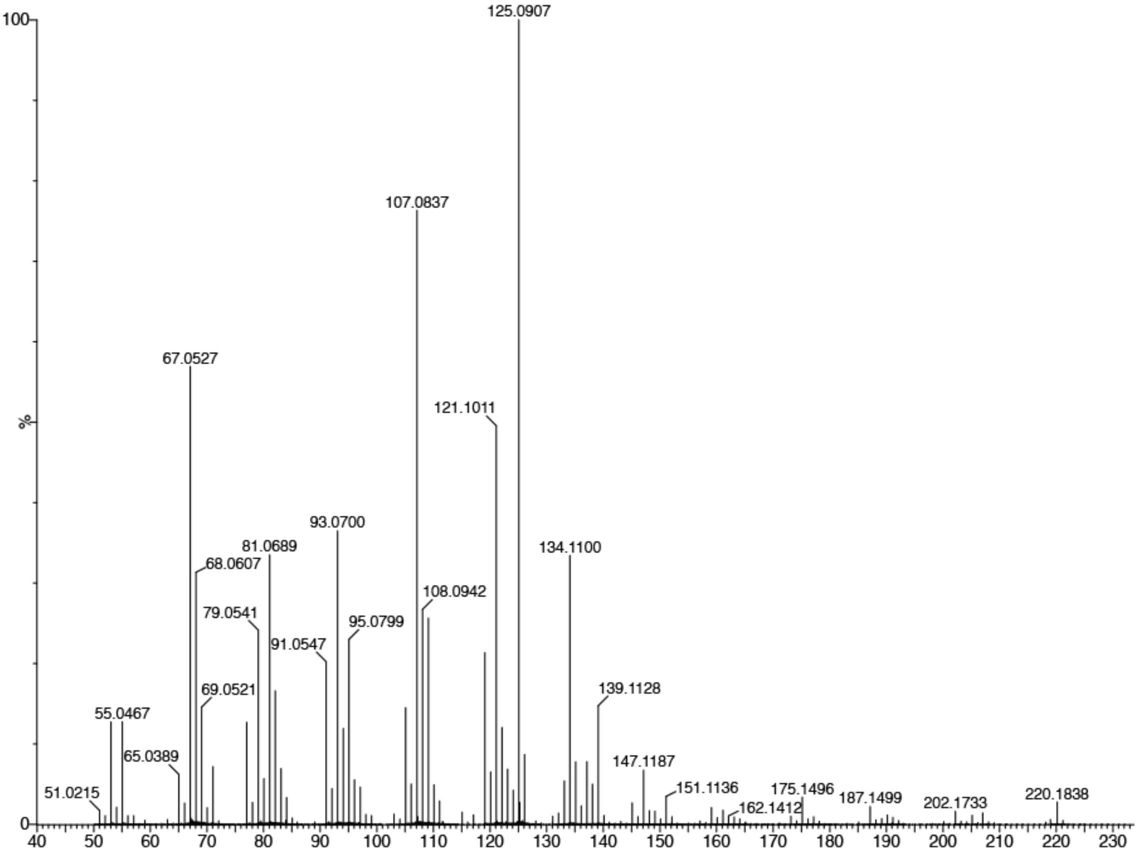

Figure S5. Mass spectrum of the compound eluting at 28.22 min in the gas-chromatogram from the incubation of **8** with GAS.

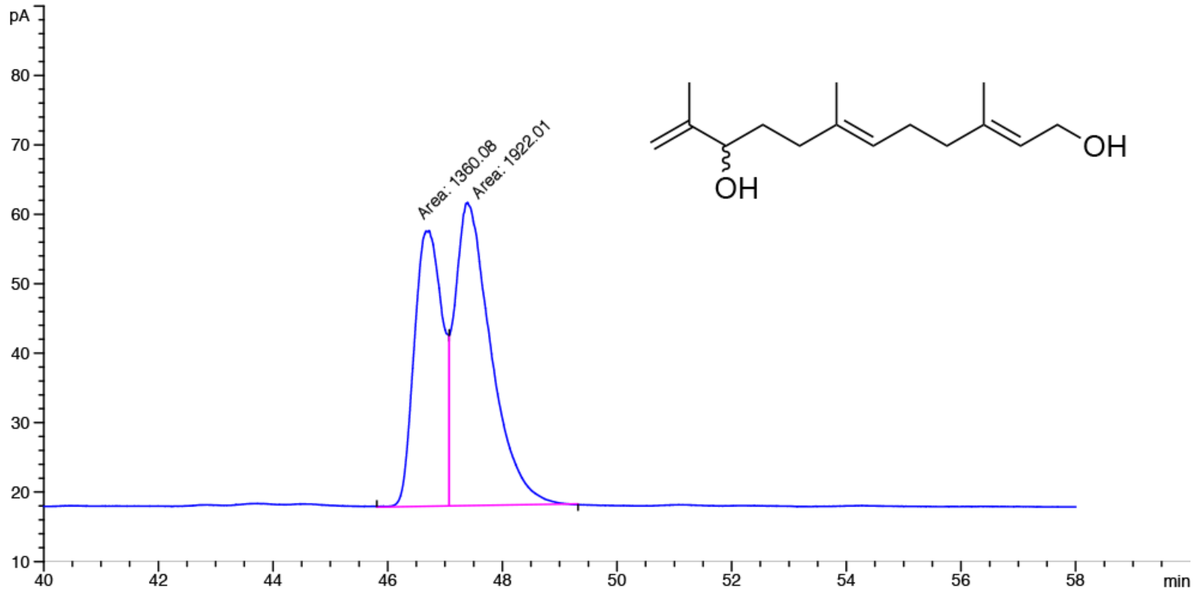

Figure S6. Gas chromatogram (FID) of. the diol precursor on a chiral stationary phase.

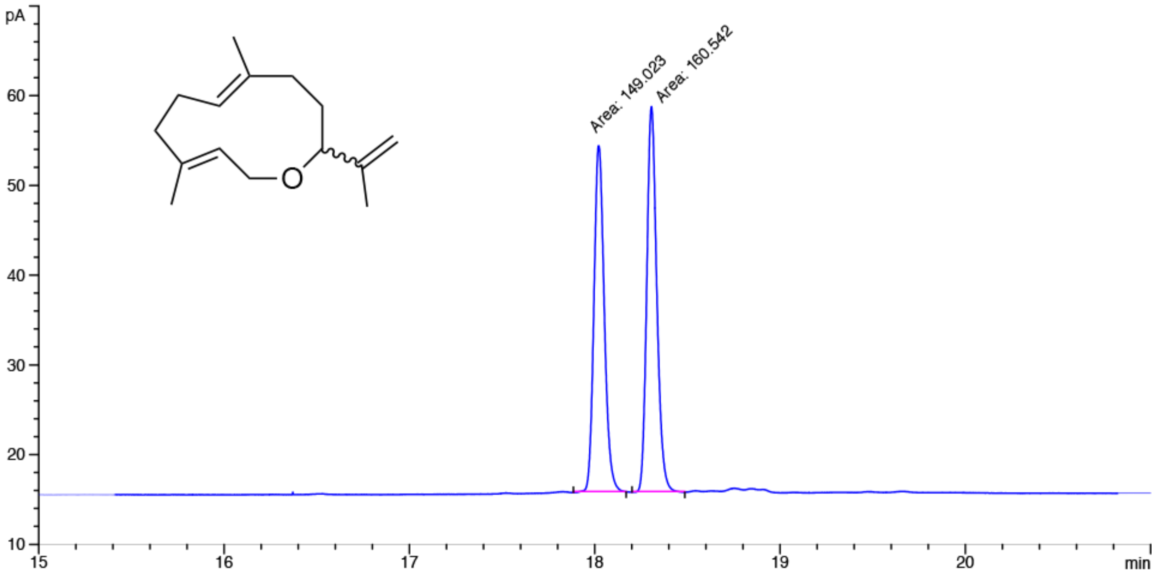

Figure S7. Gas chromatogram (FID) of racemic cyclic ether **10** on a chiral stationary phase

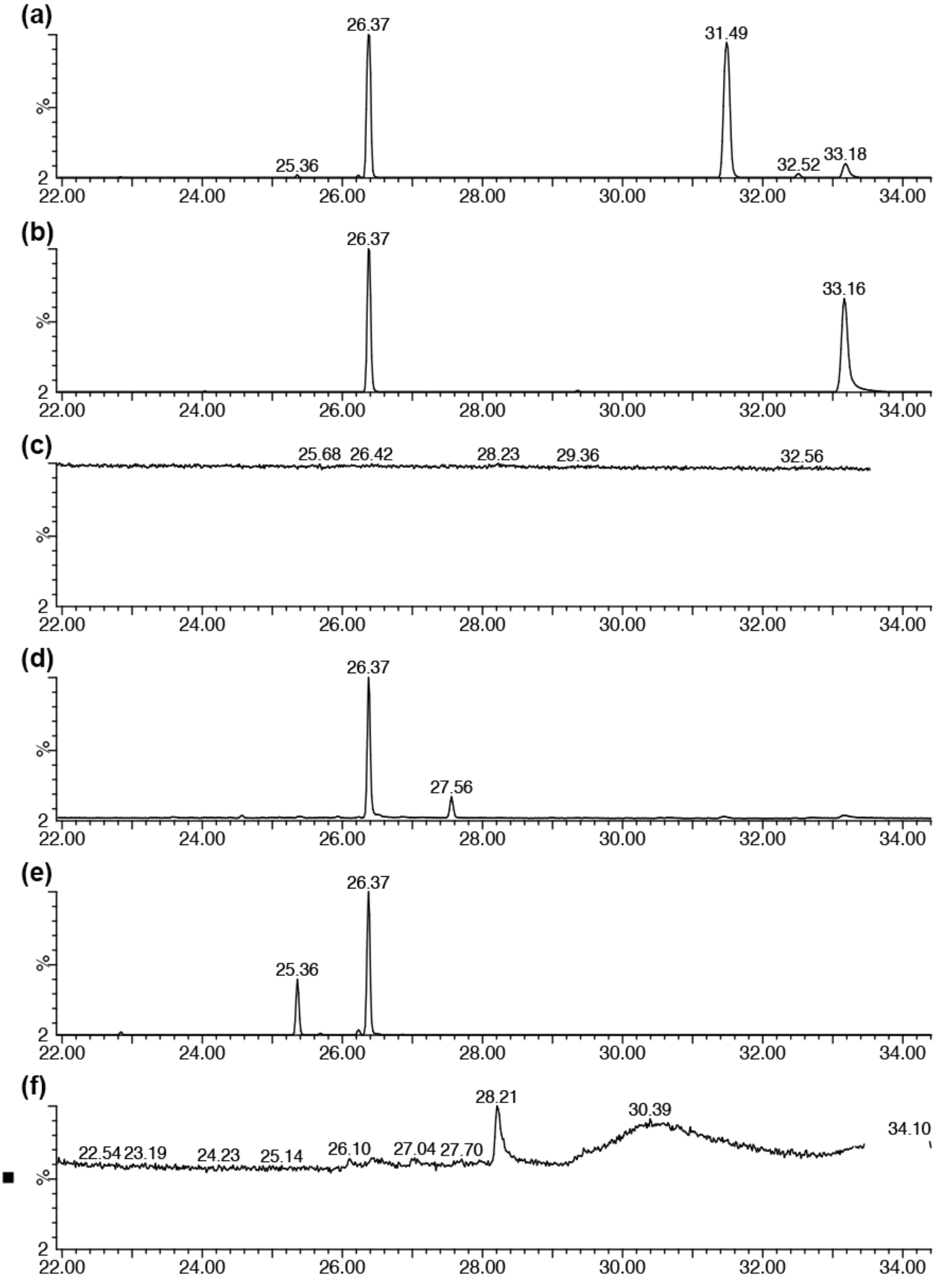

Figure S8. Total ion chromatograms of the pentane extractable products arising from: a) from the incubation of **7** with aristolochene synthase from *Penicillium roqueforti*, b) the incubation of **8** with aristolochene synthase from *Penicillium roqueforti,* c) from the incubation of **7** with (+)-δ-cadinene synthase from *Gossypium arboreum*, d) from the incubation of **8** with (+)-δ-cadinene synthase from *Gossypium arboreum*, e) the incubation of **7** with amorphadiene synthase from *Artemisia annua*, f) the incubation of **8** with amorphadiene synthase from *Artemisia annua*.

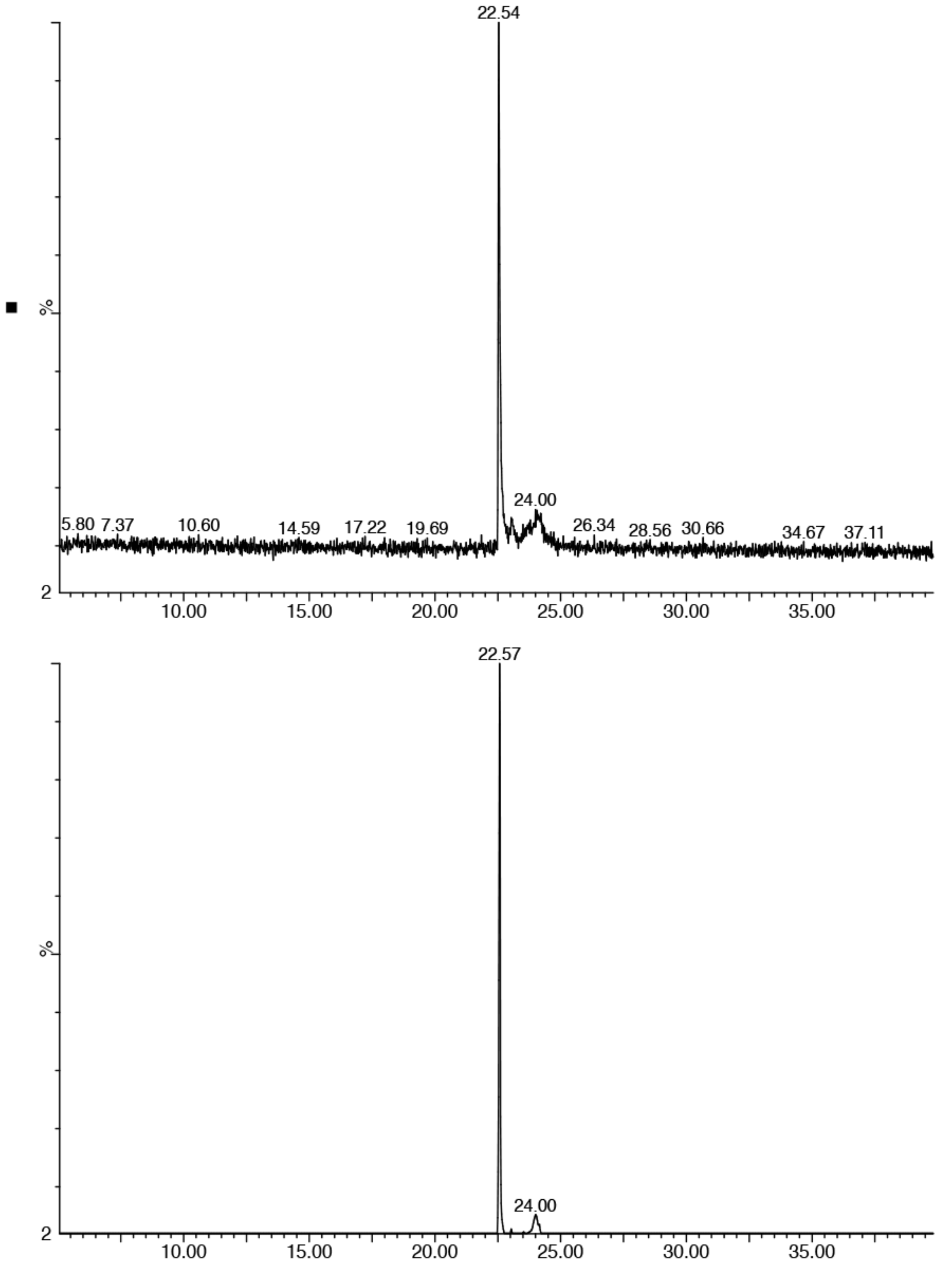

Figure S9. Top: Total ion chromatogram of the pentane extractable products from the incubation of **8** (0.3 mM) with alkaline phosphatase (EC 3.1.3.1, 30 μM) in incubation buffer (50 mM Tris, 5 mM $MgCl_2$, 5 mM βME, pH 8.0). Bottom: Total ion chromatogram of 10,11-epoxy-farnesol

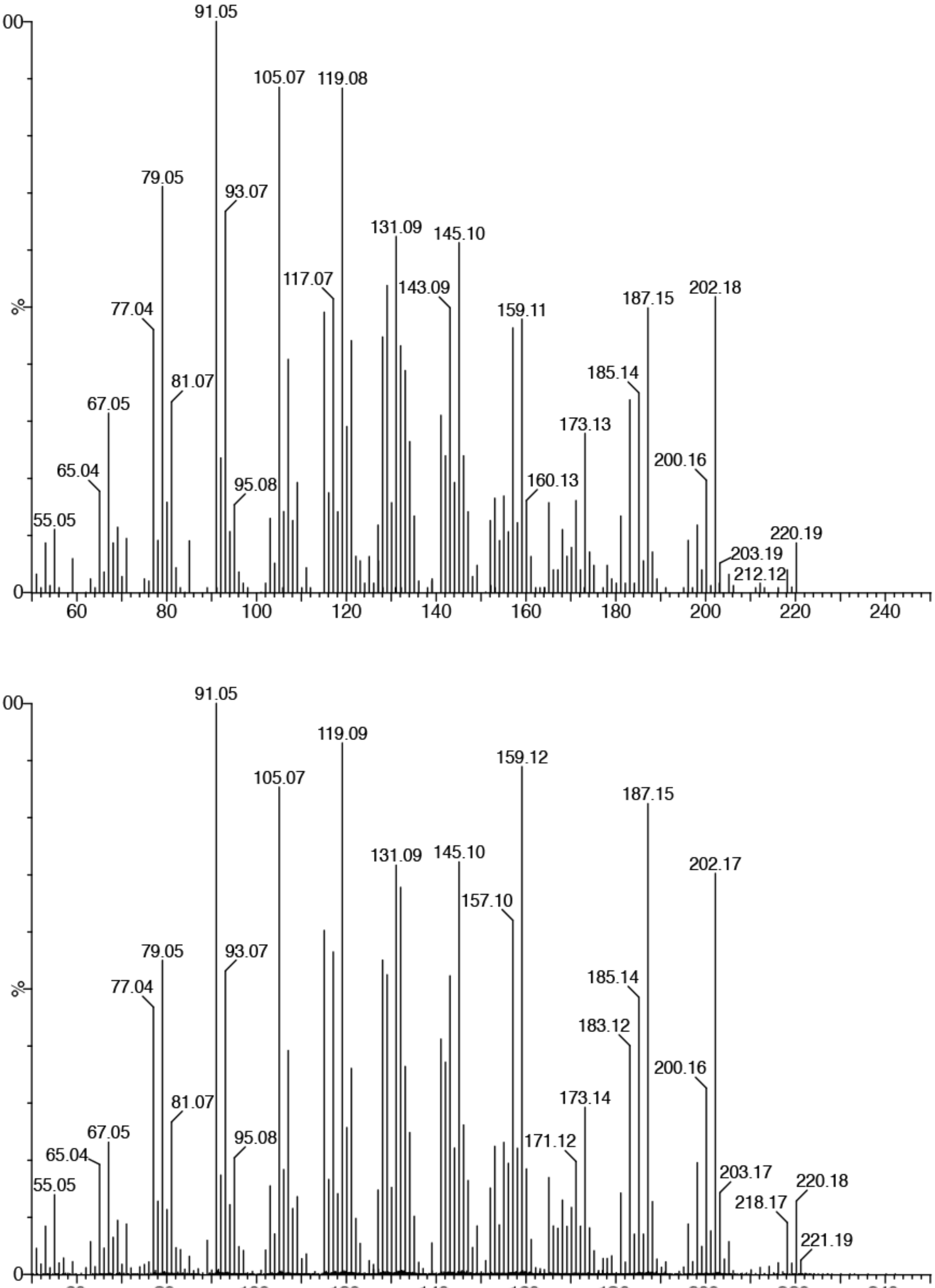

Figure S10. Top: Mass spectrum of the compound eluting at 22.54 min in the gas-chromatogram from the incubation of **8** with alkaline phosphatase. Bottom: Mass spectrum of the compound eluting at 22.57 min in the gas-chromatogram from 10,11-epoxy-farnesol

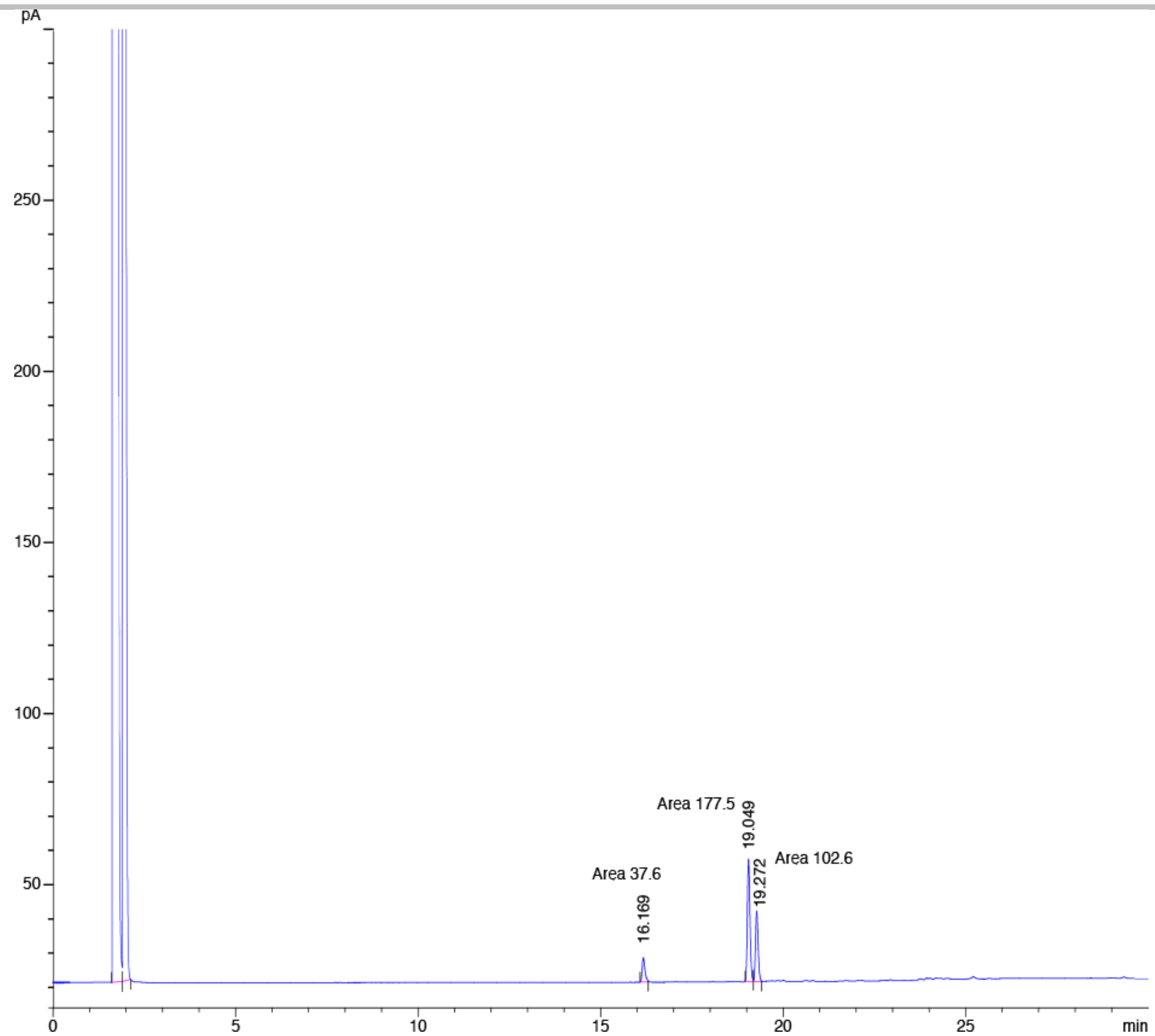

Figure S11. Gas chromatogram (FID) of the pentane extractable products arising from the incubation of **8** with GdolS carried out in the flow segmented system on a chiral stationary phase. The compound eluting at 16.169 min is the internal standard α-humulene. The compounds eluting at 19.049 min and 19.272 min correspond to the two enantiomers of cyclic ether **10**.

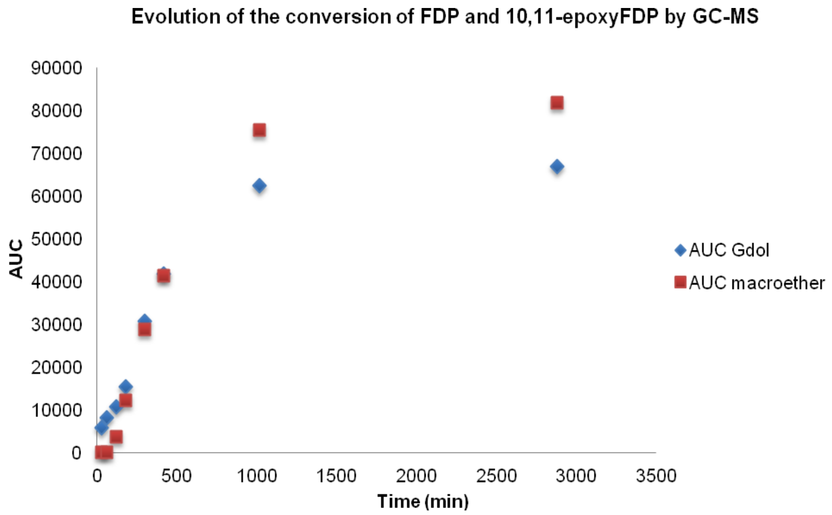

Figure S12. Relative quantities of products in a competitive assay of **8** (0.125 mM) and FDP (**1**, 0.025 mM) with GdolS as judged by relative integration of product peaks in the gas chromatogram of the pentane extractable products.

7. NMR data and spectra

Table S1. Proton NMR spectroscopic assignment of **10** at -50 °C and +50 °C

Assignment	-50 °C major conformer[a]	-50 °C minor conformer[a]	+50°C[a]
CH_2-1	3.81 – 3.68 (m)	AB part of ABX system<br>3.99 (dd, $^2J_{HH}$ = 12.0, $^3J_{HH}$ = 10.5) and 3.50 (dd, $^2J_{HH}$ = 12.5, $^3J_{HH}$ = 5.0)	AB part of ABX system<br>3.89 (dd, $^2J_{HH}$ = 12.3, $^3J_{HH}$ = 7.0) and 3.70 (dd, $^2J_{HH}$ = 9.0, $^3J_{HH}$ = 7.0)
CH-2	5.19 (dd, $^3J_{HH}$ = 10.0, $^3J_{HH}$ = 6.0)	X part of ABX system<br>5.48 (dd, $^3J_{HH}$ = 10.5, $^3J_{HH}$ = 5.0)	X part of ABX system<br>5.32 (dd, $^3J_{HH}$ = 9.0, $^3J_{HH}$ = 7.0)
CH_2-4	2.46 – 1.90 (m)	2.46 – 1.90 (m)	2.32 – 2.02 (m)
CH_2-4	2.46 – 1.90 (m)	2.46 – 1.90 (m)	2.32 – 2.02 (m)
CH_2-5	2.46 – 1.90 (m)	2.46 – 1.90 (m)	2.32 – 2.02 (m)
CH-6	4.99 – 4.89 (m)	4.99 – 4.89 (m)	4.98 (t, $^3J_{HH}$ = 7.0)
CH_2-8	2.46 – 1.90 (m)	2.46 – 1.90 (m)	2.32 – 2.02 (m)
CH_2-9	1.61 – 1.45 (m)	1.61 – 1.45 (m)	1.68 – 1.52 (m)
CH-10	3.26 (d, $^3J_{HH}$ = 9.5)	3.26 (d, $^3J_{HH}$ = 9.5)	3.51 (m)
CH_2-12	4.87 – 4.68 (m)	4.87 – 4.68 (m)	4.88 – 4.84 (m) and<br>4.84 – 4.79 (m)
CH_3-13	1.68 (s)	1.68 (s)	1.70 (s)
CH_3-14	1.49 (s)	1.54 (s)	1.54 (s)
CH_3-15	1.56 (s)	1.66 (s)	1.64(s)

[a] All resonances are δ_H (400 MHz, $CDCl_3$) in ppm. Entries are chemical-shift followed by multiplicity and coupling constants (Hz).

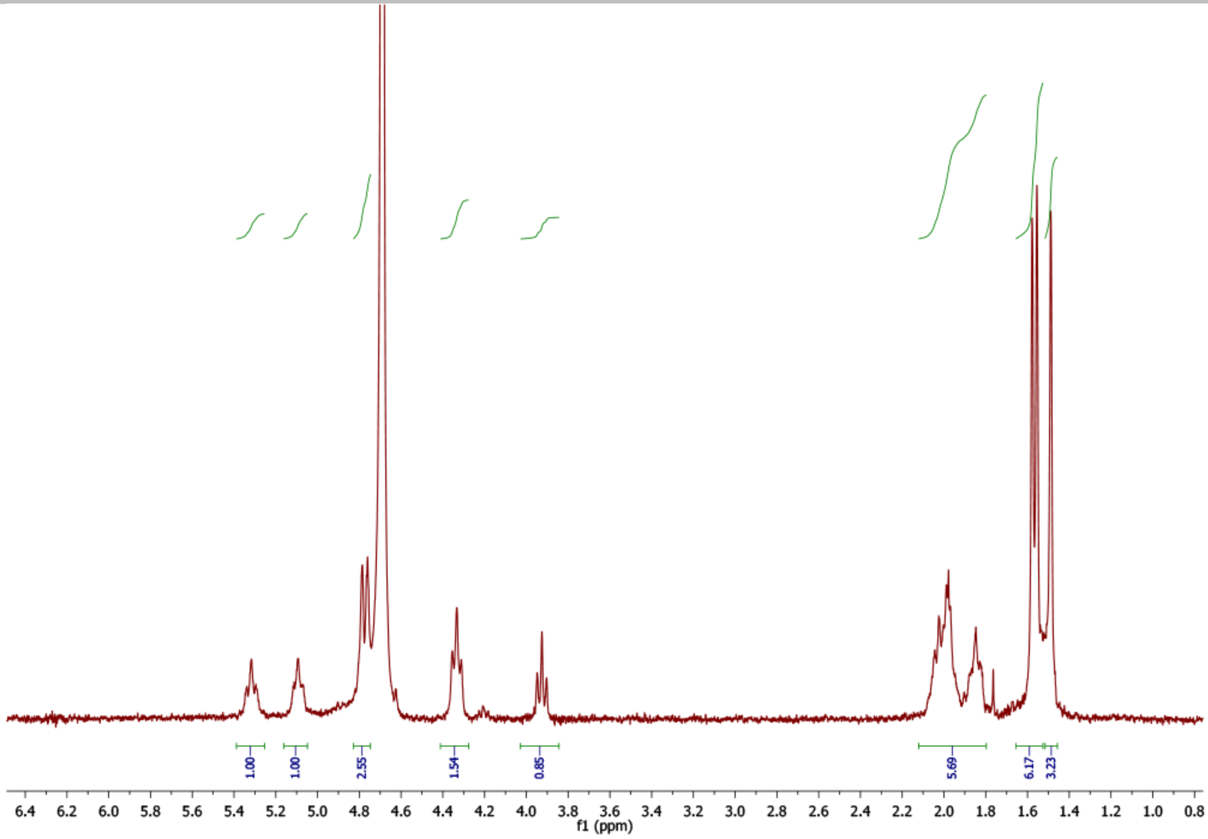

Figure S13. ^{1}H NMR spectrum (400 MHz, D_2O) of FDP analogue **7.**

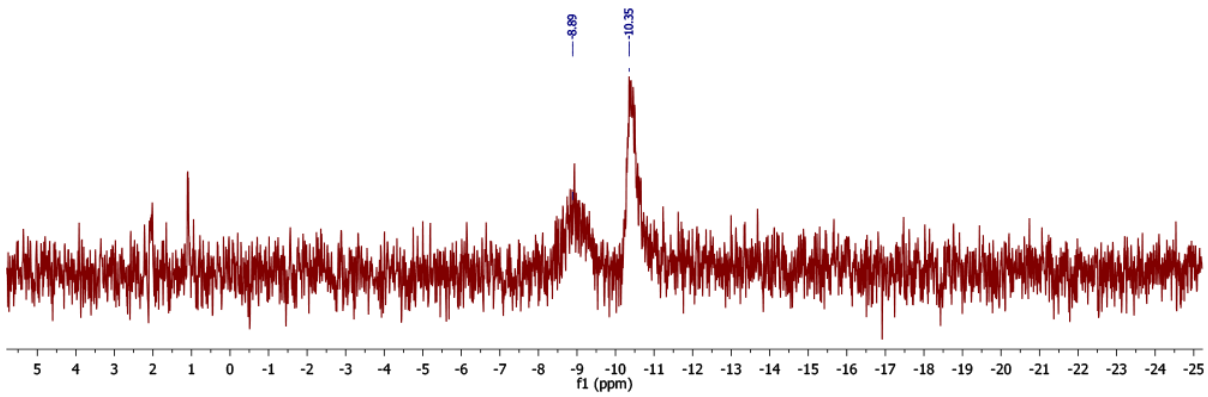

Figure S14. ^{31}P NMR spectrum (161 MHz, D_2O) of allylic alchohol-FDP analogue **7**.

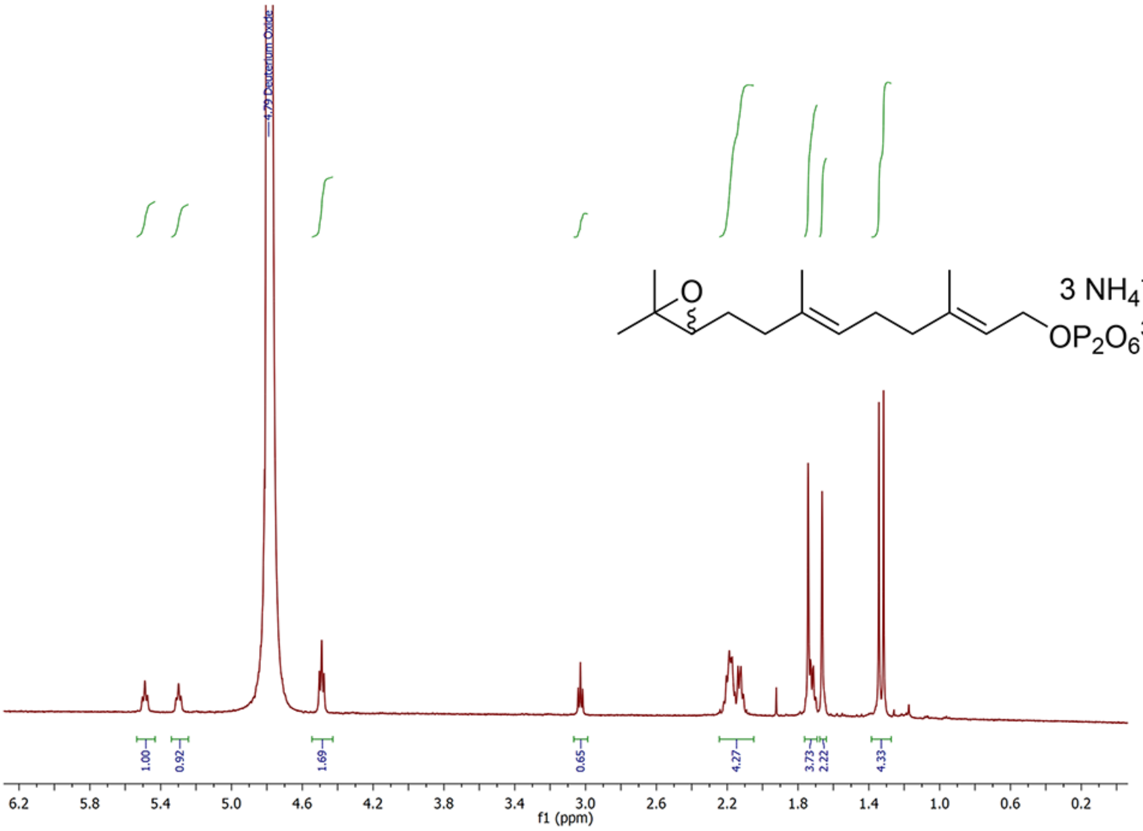

Figure S15. ^{1}H NMR spectrum (400 MHz, D_2O) of FDP analogue **8**.

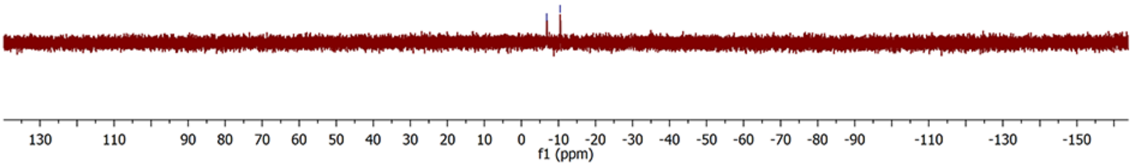

Figure S16. ^{31}P NMR spectrum (161 MHz, D_2O) of epoxy-FDP analogue **8**.

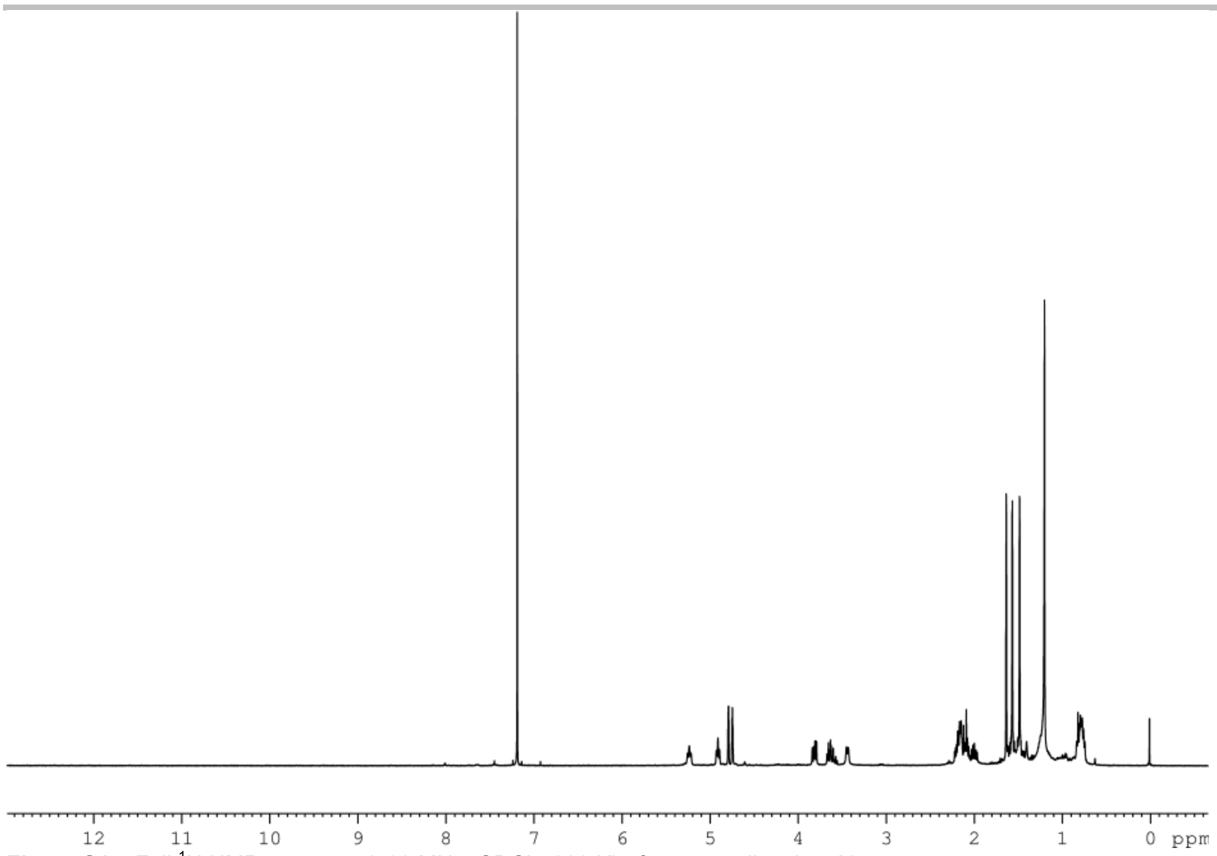

Figure S17. Full ^{1}H NMR spectrum (400 MHz, $CDCl_3$, 298 K) of macrocyclic ether **10**.

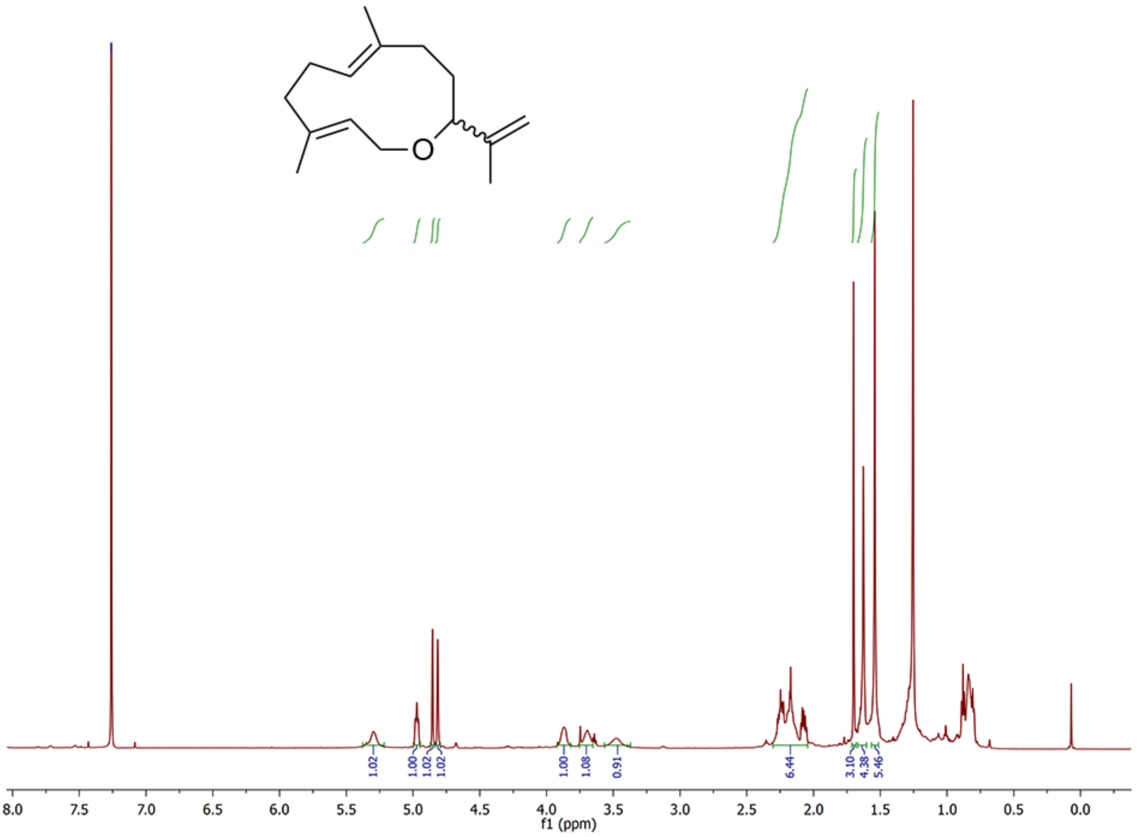

Figure S18. ^{1}H NMR spectrum (400 MHz, $CDCl_3$, 298 K) of macrocyclic ether **10**.

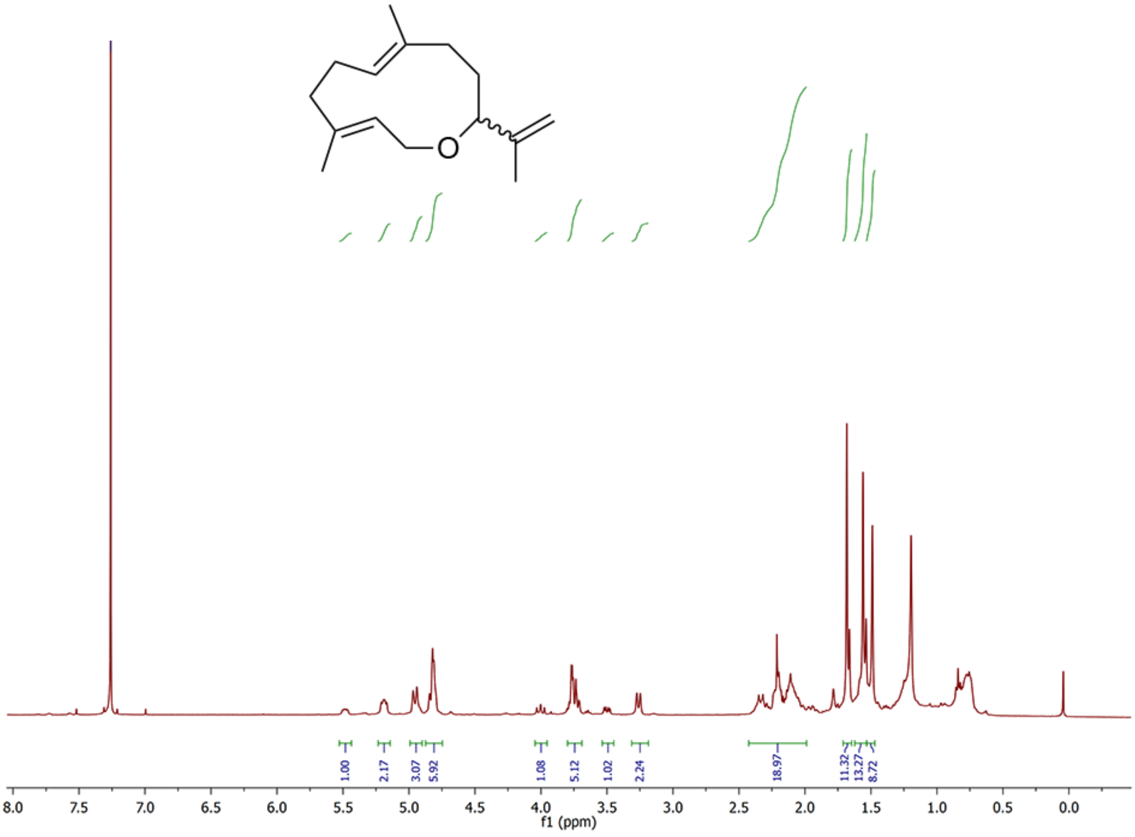

Figure S19. ^{1}H NMR spectrum (400 MHz, $CDCl_3$, 223 K) of macrocyclic ether **10**.

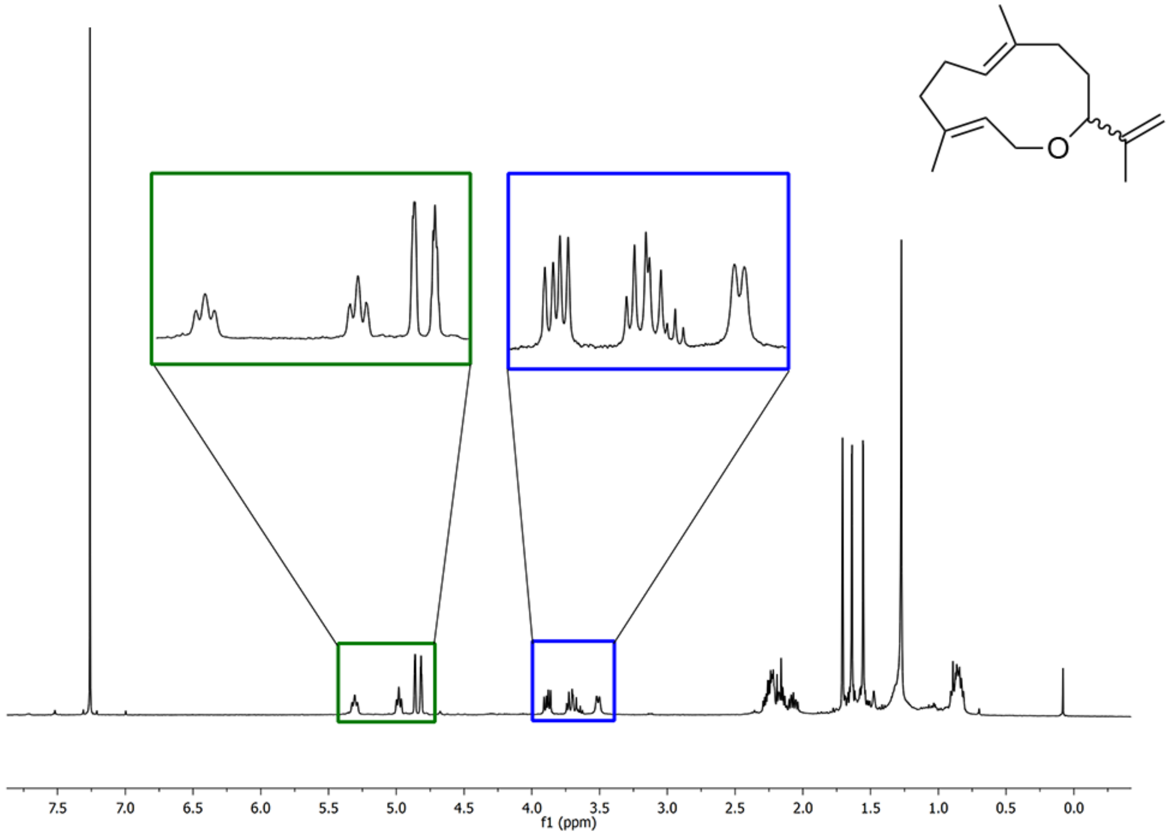

Figure S20. ^{1}H NMR spectrum (400 MHz, $CDCl_3$, 323 K) of macrocyclic ether **10**. Left green inset shows expanded view from δ_H = 4.7 -5.4 ppm region. Right blue inset shows expanded view from δ_H = 3.3 - 4.0 ppm region.

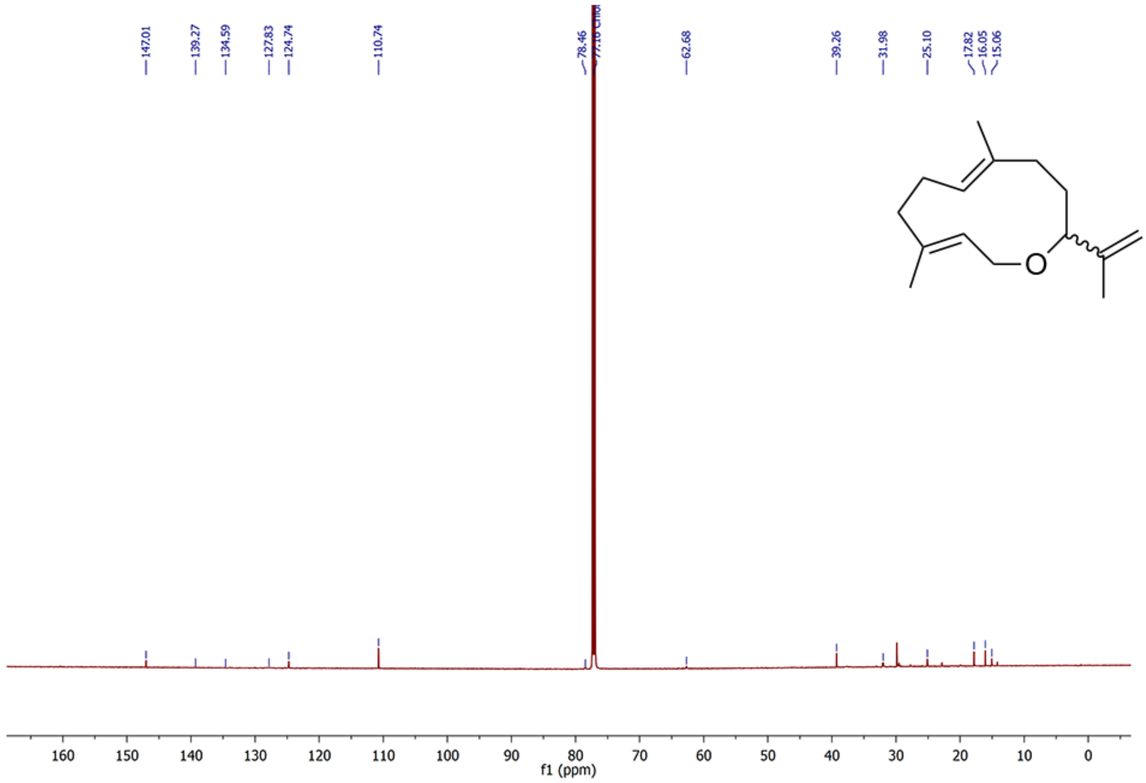

Figure S21. ^{13}C NMR spectrum (100 MHz, $CDCl_3$, 323 K) of macrocyclic ether **10**.

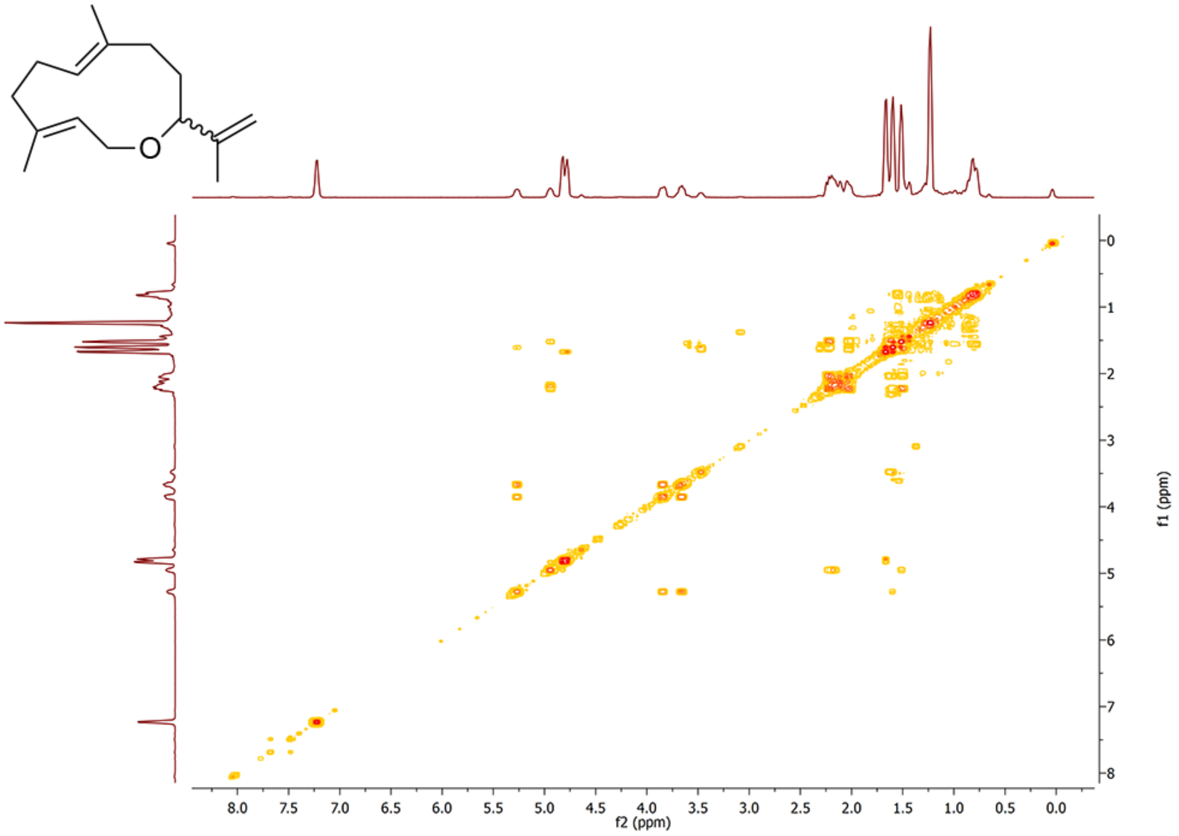

Figure S22. COSY NMR spectrum (400 MHz, $CDCl_3$, 323K) of macrocyclic ether **10**.

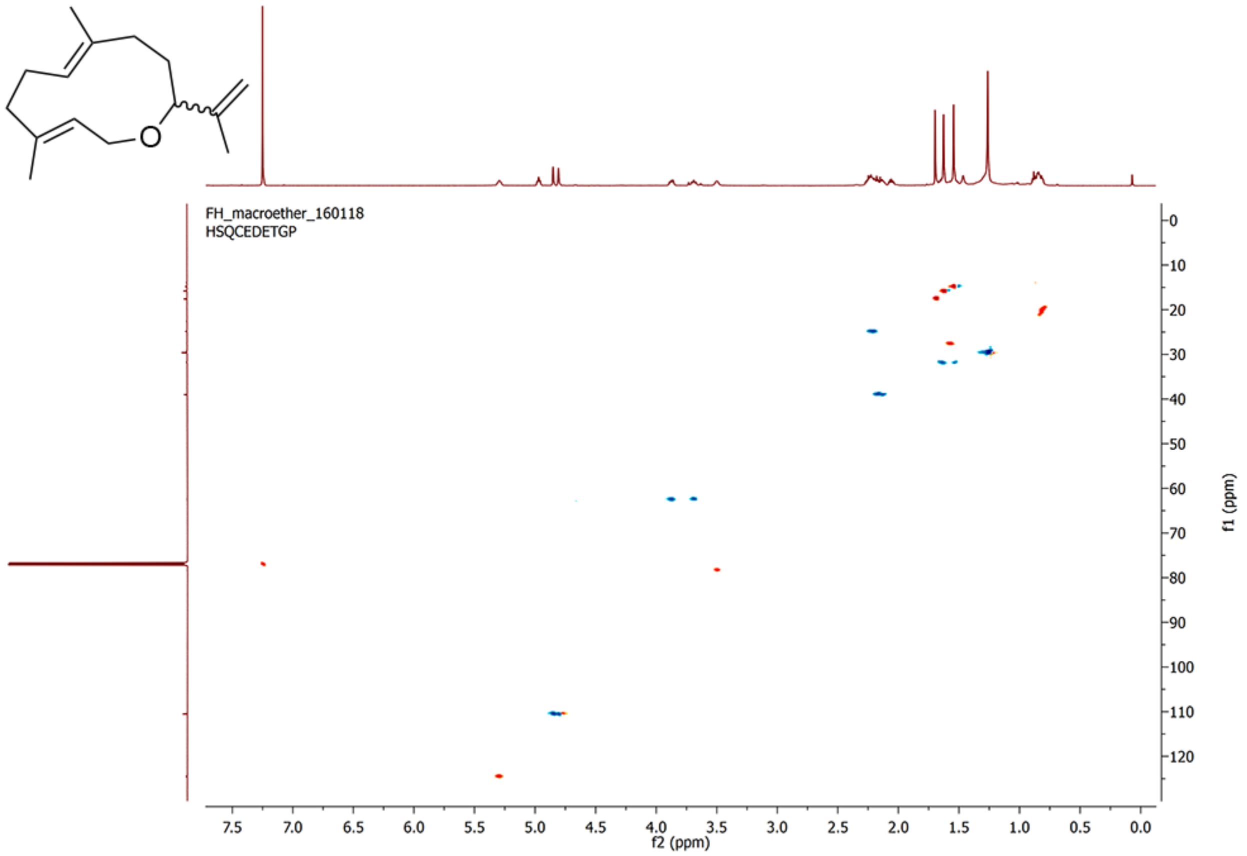

Figure S23. Edited HSQC NMR spectrum (400 MHz, $CDCl_3$, 323 K) of macrocyclic ether **10**.

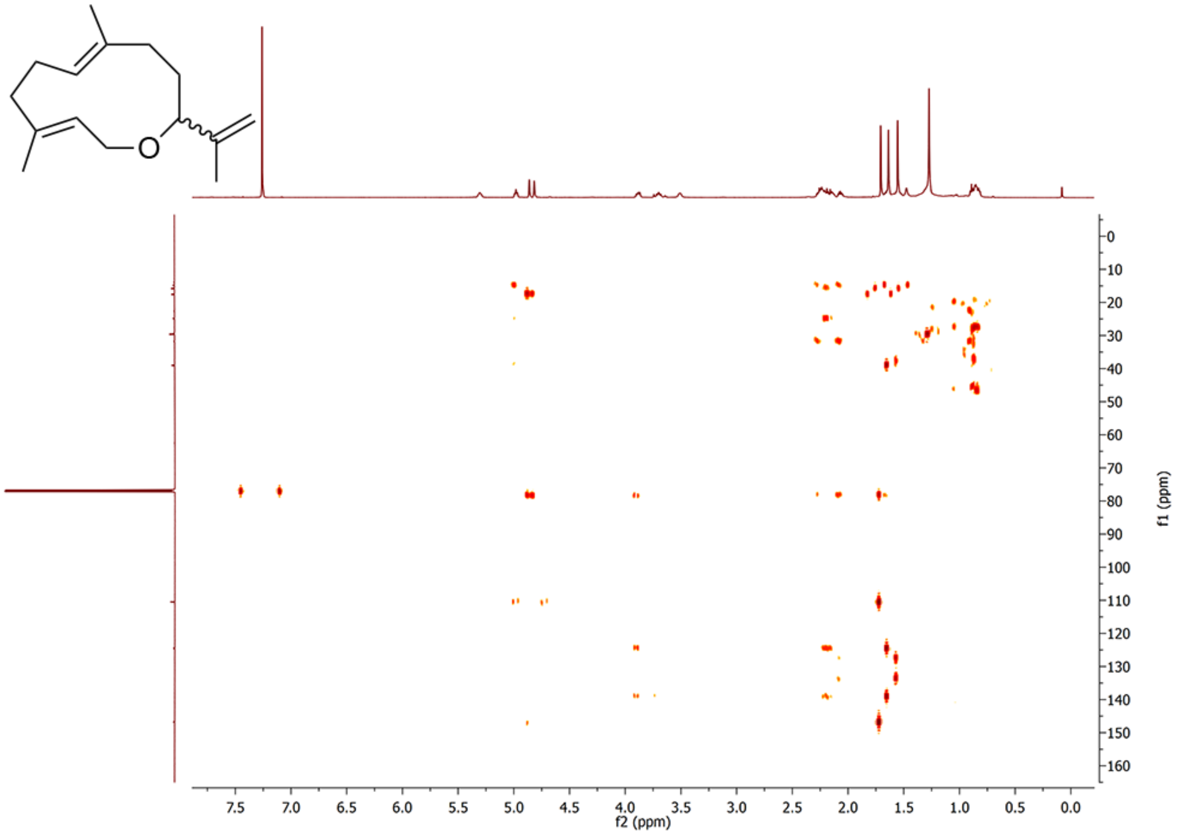

Figure S24. HMBC NMR spectrum (400 MHz, $CDCl_3$, 323 K) of macrocyclic ether **10**.

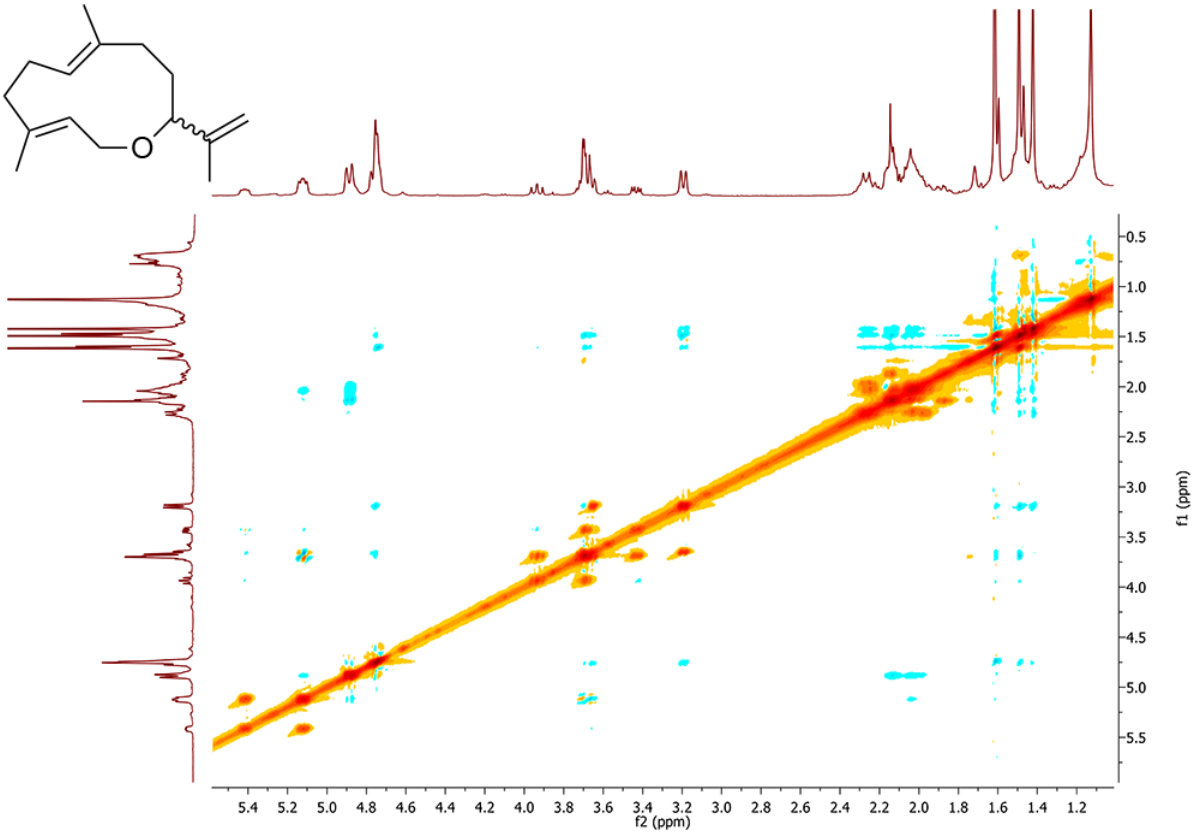

Figure S25. NOESY NMR spectrum (400 MHz, $CDCl_3$, 223 K) of macrocyclic ether **10**.

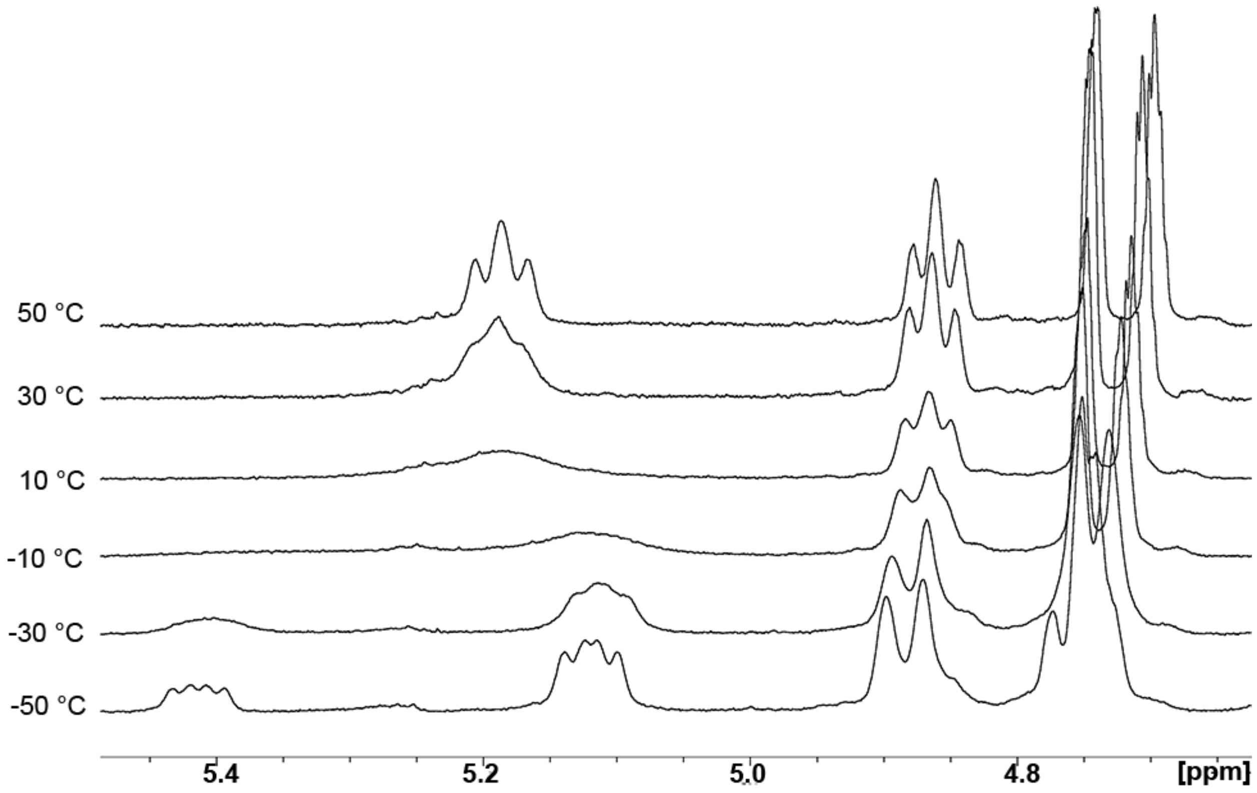

Figure S26. Stacked ^{1}H NMR spectra (400 MHz, $CDCl_3$) from -50 °C to 50 °C of macrocyclic ether **10**, alkene region.

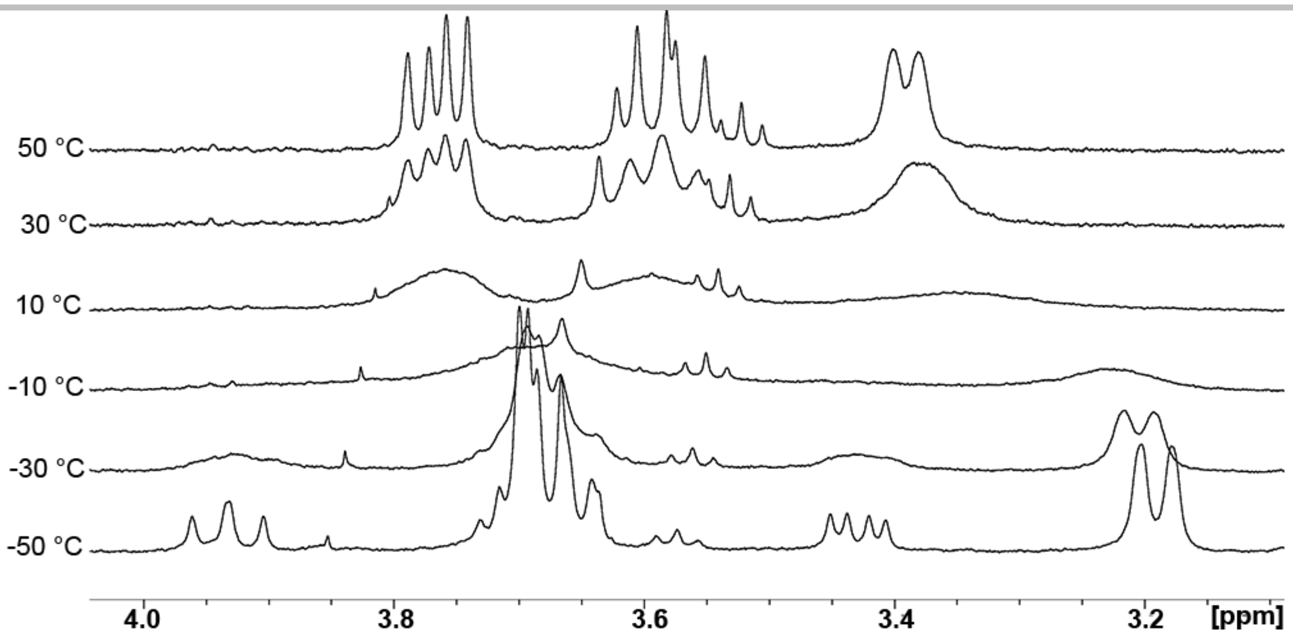

Figure S27. Stacked ^{1}H NMR spectra (400 MHz, $CDCl_3$) from -50 °C to 50 °C of macrocyclic ether **10**, δ_H = 3.1 – 4.0 ppm region.

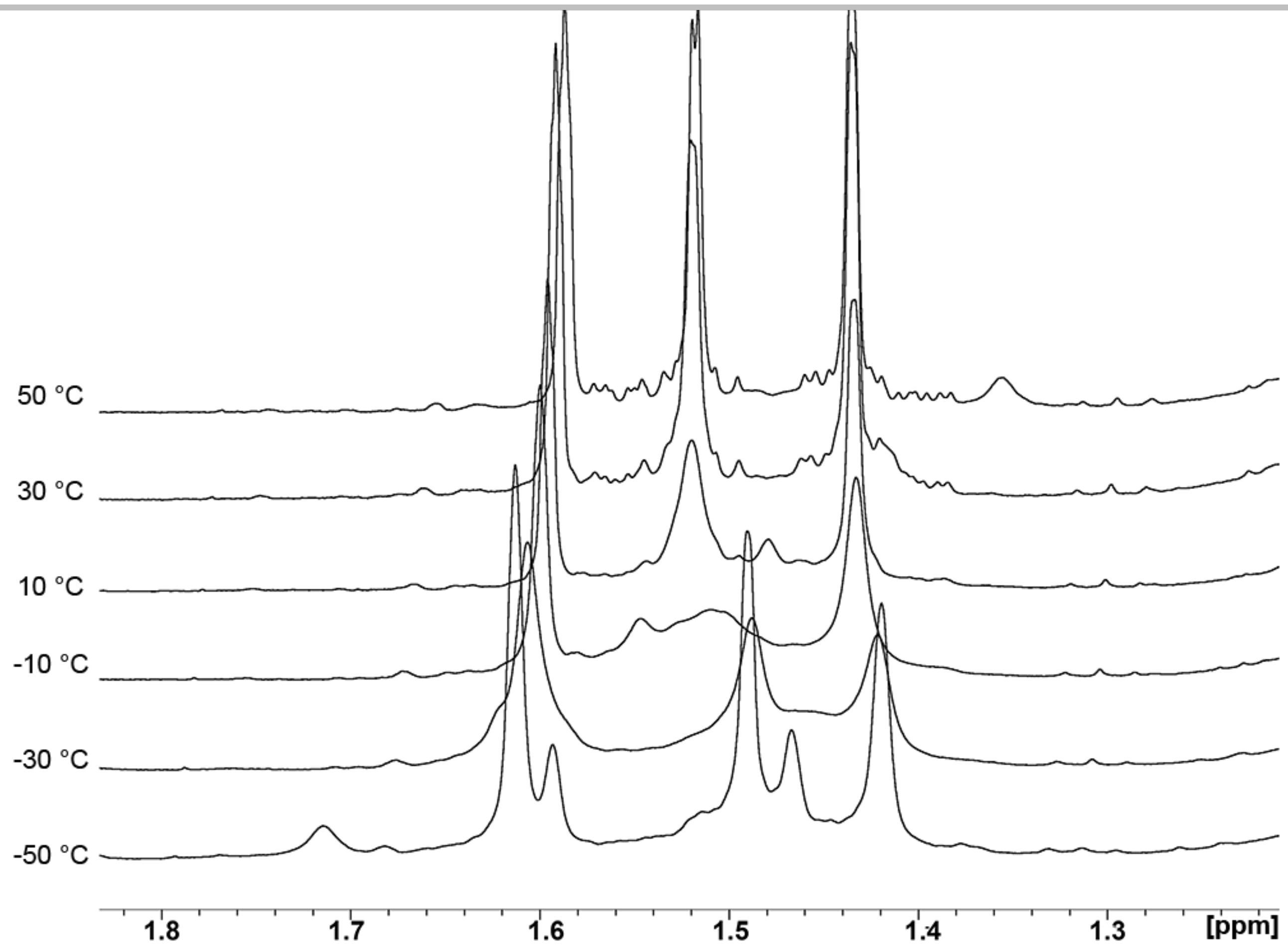

Figure S28. Stacked ^{1}H NMR spectra (400 MHz, $CDCl_3$) from -50 °C to 50 °C of macrocyclic ether **10**, alkyl region.

8. References

[1] O. Cascón, S. Touchet, D. J. Miller, V. Gonzalez, J. A. Faraldos, R. K. Allemann, *Chem. Commun.* **2012**, *48*, 9702-9704.

[2] M. M. Bradford, *Anal. Biochem.* **1976**, *72*, 248-254.

[3] C. Nakano, F. Kudo, T. Eguchi, Y. Ohnishi, *ChemBioChem* **2011**, *12*, 2271-2275.

[4] D. J. Grundy, M. Chen, V. Gonzalez, S. Leoni, D. J. Miller, D. W. Christianson, R. K. Allemann, *Biochemistry* **2016**, *55*, 2112-2121.

[5] a) J. A. Faraldos, V. González, A. Li, F. Yu, M. Köksal, D. W. Christianson, R. K. Allemann, *J. Am. Chem. Soc.* **2012**, *134*, 20844-20848; b) M. Demiray, X. Tang, T. Wirth, J. A. Faraldos, R. K. Allemann, *Angew. Chem. Int. Edit.* **2017**, *56*, 4347-4350; c) M. Loizzi, V. González, D. J. Miller, R. K. Allemann, *ChemBioChem* **2018**, *19*, 100-105; d) D. J. Miller, F. Yu, R. K. Allemann, *ChemBioChem* **2007**, *8*, 1819-1825.

[6] a) V. J. Davisson, A. B. Woodside, T. R. Neal, K. E. Stremler, M. Muehlbacher, C. D. Poulter, *J. Org. Chem* **1986**, *51*, 4768-4779; b) A. B. Woodside, Z. Huang, C. D. Poulter, *Org. Synth.* **1993**, *66*, 211-219.

[7] D. E. Cane, R. Iyengar, M.-S. Shiao, *J. Am. Chem. Soc.* **1981**, *103*, 914-931.

[8] a) M.-R. Cha, C.-W. Choi, J.-Y. Lee, Y.-S. Kim, G.-H. Yon, S.-U. Choi, S.-Y. Ryu, *B. Kor. Chem. Soc.* **2012**, *33*, 2378-2380; b) D. Xia, Y. Du, Z. Yi, H. Song, Y. Qin, *Chem. – A Eur J.* **2013**, *19*, 4423-4427.

[9] a) O. Cascón, G. Richter, R. K. Allemann, T. Wirth, *ChemPlusChem* **2013**, *78*, 1334-1337; b) X. Tang, R. K. Allemann, T. Wirth, *Eur. J. Org. Chem.* **2017**, *2*, 414-418.
